# Assessing dental students’ knowledge of panoramic radiographs and the importance of normal anatomy education

**DOI:** 10.1186/s12909-025-07829-w

**Published:** 2025-08-30

**Authors:** Yoon Joo Choi, Kug Jin Jeon, Chena Lee, Sang-Sun Han

**Affiliations:** 1 Department of Oral and Maxillofacial Radiology, Yonsei University College of Dentistry, 50-1 Yonsei-ro Seodaemun-gu, Seoul, 03722 Republic of Korea; 2 Oral Science Research Center, Yonsei University College of Dentistry, Seoul, Korea

**Keywords:** Anatomy, Diagnostic Imaging, Jaw Disease, Education, Dental, Radiography, Panoramic

## Abstract

**Background:**

This study aimed to investigate dental students’ knowledge of normal anatomical structures and their competency in diagnosing jaw lesions using panoramic radiographs, as well as to compare diagnostic accuracy among three groups with different levels of anatomical knowledge.

**Methods:**

A computer-based test, consisting of 50 panoramic radiographs (10 depicting normal anatomical structures and 40 showing jaw lesions), was conducted on November 8, 2023, at Yonsei Dental University. The 40 jaw lesions were classified into four categories: cyst, benign tumor, inflammation or malignancy, and other bone lesion. The mean score for the 10 anatomical structure questions and the mean accuracy rate for diagnosing the 40 jaw lesions were calculated. Based on their scores on the anatomical structure questions, 125 students were divided into three groups (upper, middle, and lower). The accuracy rates for diagnosing jaw lesions among these groups were statistically analyzed using the Kruskal-Wallis test (*p* = 0.05).

**Results:**

Among all students, the mean score for normal anatomical structures was 5.99 out of 10, and the mean accuracy rate for diagnosing jaw lesions was 44.8%. In the analysis of jaw lesions, the four categories exhibited significant differences in accuracy rates: cyst (53.8%), benign tumor (47.7%), inflammation or malignancy (45.0%), and other bone lesion (32.7%). The three groups based on anatomical structure scores showed significantly different accuracy rates for diagnosing jaw lesions (*p* < 0.05). The upper group, with the highest anatomical structure scores, achieved an accuracy rate of 54.5%, outperforming the other groups.

**Conclusions:**

Knowledge of anatomical structures enhances the ability to diagnose jaw lesions using panoramic radiographs. These findings underscore the importance of anatomical education in dental curricula to improve diagnostic accuracy.

**Supplementary Information:**

The online version contains supplementary material available at 10.1186/s12909-025-07829-w.

## Background

Panoramic radiograph (PR) is widely used in dental diagnosis and treatment planning due to its relatively low cost and radiation exposure. It is commonly employed in routine dental check-ups to evaluate the entire dentition and surrounding jaw structures [1, 2]. PRs can visualize various jaw lesions, from cysts and tumors to cancers [1–3]. This diagnostic capability makes them useful for ruling out underlying lesions before treatments for caries and periodontal disease [4–7].

However, PR has a complex geometry that differs from conventional two-dimensional images acquired from fixed X-ray tubes and detectors (e.g., chest radiography). It is a tomographic image created by the rotation of the X-ray source around the patient, resulting in a characteristic arch-shaped focal layer. This rotational motion can produce real, ghost, or double images of a single structure. The image layer may also cause unequal magnification and geometric distortion, depending on the machine and patient positioning [1, 2]. Additionally, the complex midfacial anatomy often leads to overlap between the jaws, skull bones, and soft tissues, making it difficult to identify anatomical landmarks and recognize pathological changes [2]. These factors make it challenging for students to grasp the principles of PR and interpret the images accurately [8].

Thus, PRs are not always accurately interpreted, even though this is an essential skill for dentists. Previous reports indicate that information suggesting malignancy has sometimes been missed because they can mimic simple dental problems [4, 6, 7]. For example, toothache caused by salivary gland tumors or maxillary sinus cancer is often misdiagnosed as a simple endodontic or periodontal lesion [4, 6]. Diagnostic evidence of a pathological condition on PR is usually manifested as the destruction of anatomical structures. In the absence of symptoms, these signs may be overlooked, resulting in delayed diagnosis, poorer prognosis, and reduced quality of life [4–7]. Vogel and Schulze [9] demonstrated through an eye-tracking study that dental students tended to focus only on symptomatic regions, and for accurate radiologic interpretation, they should develop more systematic scanning methods. Therefore, it is necessary to enhance dental students’ ability to detect pathological lesions on PRs even in the absence of clinical symptoms.

Many oral radiology textbooks devote significant space to describing normal anatomical structures [1, 10, 11]. Given the necessity of thoroughly understanding these structures for accurate diagnosis, several studies have assessed their knowledge level in PR interpretation [12, 13]. İlgüy et al. [12] and Maeda et al. [13] evaluated students’ understanding of more than 20 normal anatomical structures. Additionally, Azimi et al. [14] assessed the diagnostic accuracy for pathological lesions using a limited set of 13 cases and 36 students, highlighting potential challenges in PR interpretation. Furthermore, Shintaku et al. [15] showed that education on anatomical landmarks using PRs improved diagnostic outcomes of osteoporosis. They also suggested that there was a strong correlation between anatomical knowledge and the ability to identify pathological conditions. These findings support the need to reinforce anatomical education in oral radiology training.

Therefore, we hypothesized that improving dental students’ understanding of normal anatomical structures on PR could improve accuracy in diagnosing jaw lesions. This study aimed to investigate dental students’ knowledge of normal anatomical structures and their competency in diagnosing various jaw lesions using PRs. In addition, we compared the diagnostic accuracy of jaw lesions among groups of dental students classified by their scores on normal anatomical structure questions. This study provides essential baseline data for improving the quality of PR interpretation education and developing new teaching methods.

## Methods

### Competency test administration and data collection

The competency test was administered to dental students as part of the regular curriculum, and response data were retrospectively analyzed. This study was approved by the Institutional Review Board of Yonsei University Dental Hospital (IRB No. 2-2024-0080). As the test was included in the educational program, the requirement for informed consent was waived. A computer-based test was conducted on November 8, 2023, involving 125 dental students (63 fifth-year and 62 sixth-year students) at Yonsei University College of Dentistry. Because theoretical education in the oral radiology curriculum is completed before the fifth year, both fifth-year and sixth-year students were included. According to a retrospective power analysis conducted using G*Power (Version 3.1, Düsseldorf, Germany, www.psychologie.hhu.de), a sample size of 125 dental students provided a statistical power of 0.64 [16].

The test was administered using the Ubiquitous-Based Test Total Management System (UBT-TMS, NSDevil Co. LTD, Daejeon, South Korea, https://www.nsdevil.com/). PRs were presented in JPEG format, and students could freely adjust image size and contrast. All questions were provided in Korean. The test duration was 50 min considering students’ attention span and fatigue.

This competency test has been conducted annually since 2020 to assess the minimum interpretation competency of PR required for dental school graduation. Fifty questions were randomly selected from a validated question bank consisting of cases showing typical imaging features referring to the textbook [1]. Low-quality questions were excluded annually through consensus review by three board-certified oral radiologists with clinical experience: Y.J.C. (15 years), K.J.J. (23 years), and S.H. (26 years). The test was initially validated over a two-year period (2020–2021), and the cutoff for passing was set at 60%, based on the Modified Angoff method [17]. All questions used in this study was translated into English and is presented in Supplement 1.

### Test structure and contents

**Fig. 1:**
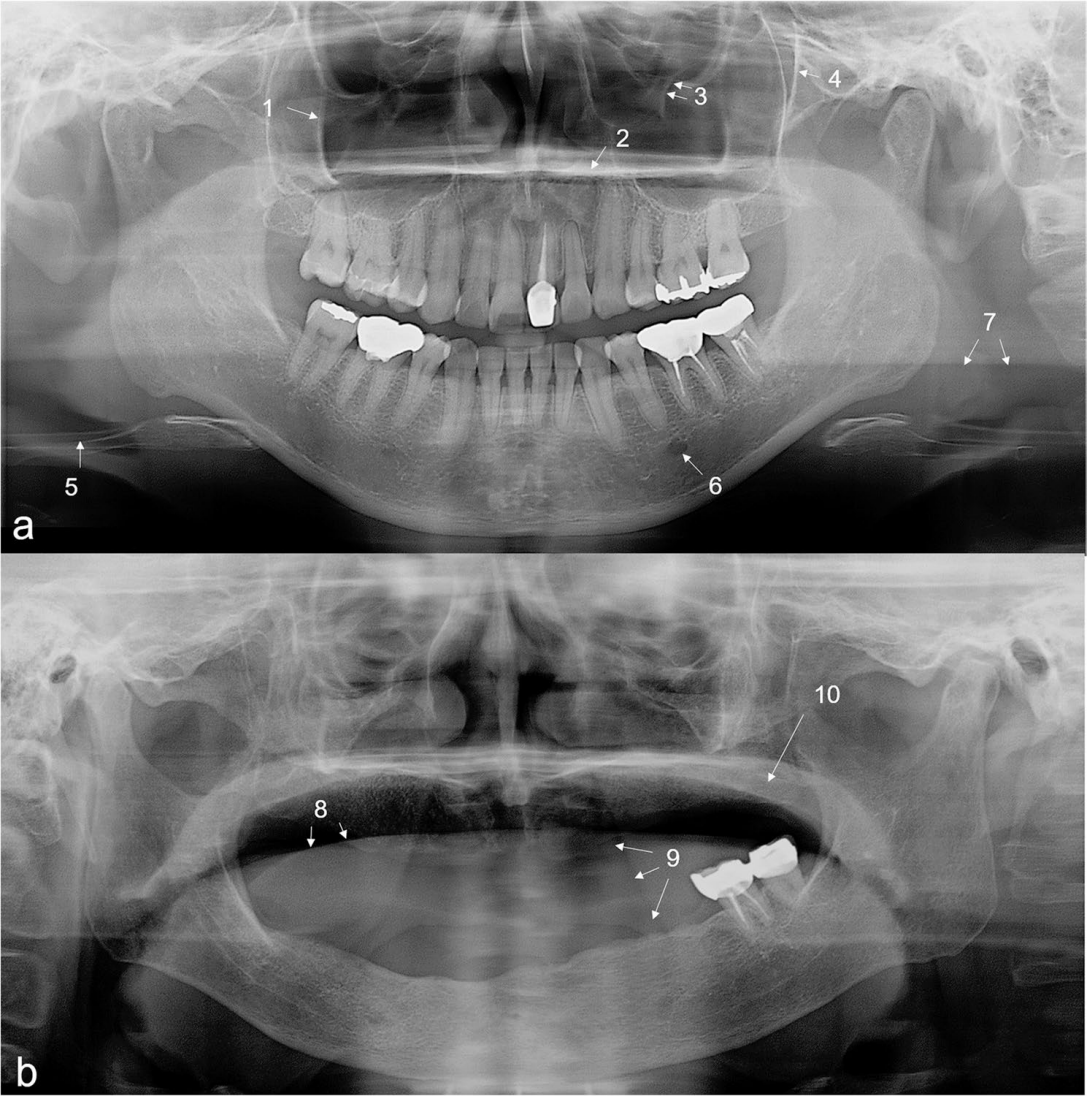
Ten questions about normal anatomical structures on two panoramic radiographs presenting bone (**a**) and soft tissue structures (**b**). 1: innominate line, 2: hard palate, 3: infraorbital canal, 4: pterygomaxillary fissure, 5: hyoid bone, 6: mental foramen, 7: ghost image of mandibular angle, 8: dorsal surface of tongue, 9: nasolabial fold, 10: soft palate

**Fig. 2:**
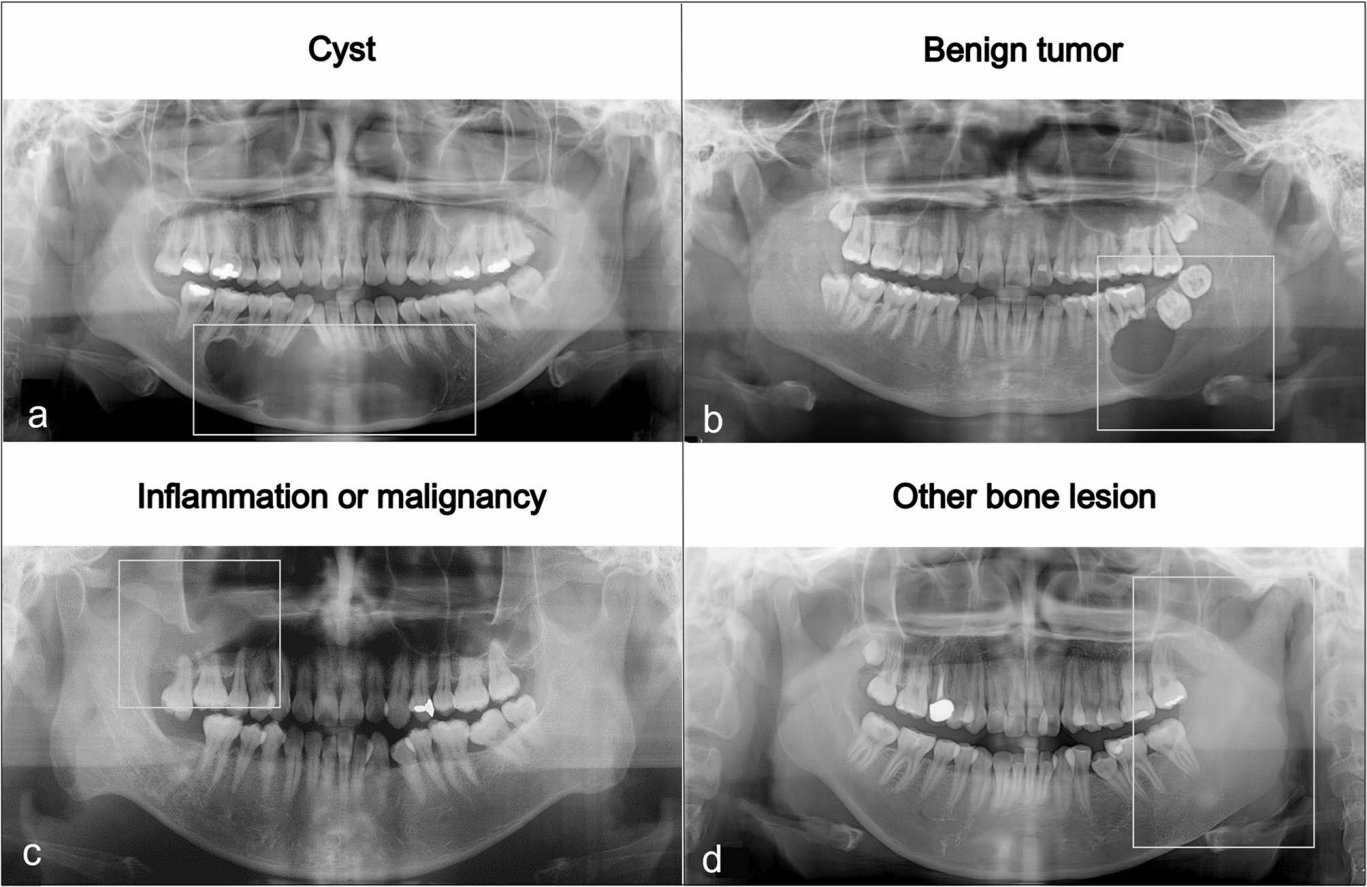
Examples of four categories of jaw lesions. Cyst (**a**), benign tumor (**b**), inflammation or malignancy (**c**), and other bone lesion (**d**). Jaw lesions are presented in white boxes on each panoramic radiograph

The test comprised 50 questions: 10 questions related to normal anatomical structures (Fig. 1) and 40 questions focused on the diagnosis of jaw lesions. Forty pathological jaw lesions were used, with 10 examples in each of the following categories: cyst, benign tumor, inflammation or malignancy, and other bone lesion (Table 1; Fig. 2). The differential diagnosis criteria for jaw lesions were based on the textbook [1], and case selection was conducted applying the following criteria, on consensus by the three oral radiologists in charge of the course.

**Table 1:** Distribution of 40 jaw lesions according to four categories

Cyst	Benign tumor	Inflammation or malignancy	Other bone lesion
Odontogenic keratocyst (4)	Adenomatoid odontogenic tumor (2)	Osteomyelitis (5)	Fibrous dysplasia (5)
Dentigerous cyst (3)	Ameloblastoma (2)	Malignancy (5)	Ossifying fibroma (2)
Nasopalatine duct cyst (1)	Odontoma (2)		Cherubism (1)
Post-operative maxillary cyst (1)	Ameloblastic fibro-odontoma (1)		Simple bone cyst (1)
Radicular cyst (1)	Odontogenic myxoma (1)		Osseous dysplasia (1)
	Central odontogenic fibroma (1)		
	Schwannoma (1)		

The numbers in parentheses indicate the number of lesions

- A single jaw lesion in each PR, either unilaterally or bilaterally, measuring ≥ 10 mm
- Representative radiographic features clearly described in the textbook [1]
- Pathological changes in the surrounding bone clearly visible on PRs
- Diagnostic histological confirmation after surgery
- Images free from artifacts or distortions that could interfere with interpretation

To simulate the clinical situation of ruling out the possibility of pathological jaw lesions prior to dental treatment, all questions were presented to students only as PRs without any clinical information. This is consistent with the goal of oral radiology education to develop students’ ability to detect lesions regardless of whether they are symptomatic. The students were asked to identify the lesion’s location and provide the most appropriate diagnosis based on the characteristic and distinctive imaging features described in the textbook [1]. For example, the key feature of a dentigerous cyst is its periphery engaging the tooth at the cementoenamel junction, whereas ameloblastoma is characterized by a uni- or multilocular radiolucency with marked cortical expansion and aggressive root resorption of adjacent teeth. In malignant lesions, only cases showing pathologic bony changes suggestive of malignancy on PR (e.g., infiltrative bone destruction, aggressive destruction of neighboring structures, floating teeth) were included. Because their histopathologic entity is not radiographically discernible, students were instructed to respond using the term “malignancy.”

### Statistical analysis

Considering the small number of questions on normal anatomical structures, total scores were calculated based on the number of correct answers. Based on these scores, students were divided into three groups: upper, middle, and lower. The mean diagnostic accuracy rate (%) for jaw lesions was calculated for each category, and differences between categories were statistically analyzed. Furthermore, the diagnostic accuracy rates for jaw lesions were compared among the three groups using the Kruskal-Wallis test (*p* = 0.05). All statistical analyses were performed using GraphPad Prism version 8.0 for Windows (GraphPad Software, Boston, Massachusetts, USA, www.graphpad.com).

## Results

Among all students, the mean score for normal anatomical structures was 5.99 out of 10. The 125 students were divided into three groups based on their scores: the lower group (score 0–3, *n* = 40), the middle group (score 4–7, *n* = 46), and the upper group (score 8–10, *n* = 39).

Overall, the mean accuracy rate for diagnosing jaw lesions was 44.8%. The accuracy rates for each category were as follows: cyst (53.8%), benign tumor (47.7%), inflammation or malignancy (45.0%), and other bone lesion (32.7%). For most pairwise comparisons between categories—except for cyst versus benign tumor and benign tumor versus inflammation/malignancy—significant differences in accuracy rates were observed (Kruskal-Wallis test, *p* < 0.05) (Table 2).

**Table 2:** Diagnostic accuracy rate for each of the four categories of jaw lesions

Category of jaw lesions	Mean $\:\pm\:\text{S}\text{D}$ (%)	Median (%)	Interquartile range [25%, 75%]	95% CI (%)
Cyst^*^	53.8$\:\:\pm\:\:$17.8	60.0	[40.0, 70.0]	50.7–57.0
Benign tumor^*§^	47.7$\:\:\pm\:\:$17.2	50.0	[40.0, 60.0]	44.7–50.7
Inflammation or malignancy^§^	45.0$\:\:\pm\:\:$22.5	50.0	[30.0, 60.0]	41.1–49.0
Other bone lesion	32.7$\:\:\pm\:\:$21.8	30.0	[15.0, 50.0]	28.9–36.6

*SD* standard deviation, *CI* confidence interval

Significant differences were observed among the four categories, except for the two comparisons indicated by * and §, according to the Kruskal–Wallis test (*p* = 0.05)

The mean accuracy rates for diagnosing jaw lesions in the lower, middle, and upper groups were 35.4%, 44.8%, and 54.5%, respectively (Table 3). Statistically significant differences in diagnostic accuracy rates were observed among the three groups (Kruskal-Wallis test, *p* < 0.05). The upper group, which scored highest on the anatomical structure questions, demonstrated a higher accuracy rate on the 40 jaw lesion questions compared to the other groups.

**Table 3:** Diagnostic accuracy rate of jaw lesions according to the knowledge level of anatomical structures

Group	Number of students	Mean $\:\pm\:\text{S}\text{D}$ (%)	Median (%)	Interquartile range [25%, 75%]	95% CI (%)
Lower group	39	35.4 ± 14.6	32.5	[47.5, 62.5]	30.7–40.0
Middle group	46	44.8 ± 12.3	45.0	[35.0, 53.25]	41.2–48.5
Upper group	40	54.5 ± 12.3	55.0	[25.0, 45.0]	50.5–58.5

Significant differences were observed among the three groups based on the Kruskal–Wallis test (*p* = 0.05)

*SD* standard deviation, *CI* confidence interval

## Discussion

We assessed dental students’ knowledge of normal anatomical structures and their competency in diagnosing jaw lesions using PRs. We also compared diagnostic accuracy rate among three groups based on anatomical knowledge levels. Students with greater knowledge of normal anatomical structures demonstrated higher diagnostic accuracy, although the overall mean did not exceed 50%. This retrospective study suggests that the level of understanding of normal anatomical structures affects the diagnosis of pathological jaw lesions, highlighting the importance of normal anatomy education within oral radiology.

Several studies have evaluated the understanding of normal anatomical structures and the recognition of pathological conditions on PR [12–14, 18], as summarized in Table 4. İlgüy et al. [12] assessed students’ knowledge of 26 anatomical structures using both PRs and periapical images. Also, they reported that third-year students—who had recently received education on anatomical structures—achieved the highest accuracy rate of 59.62%. Similarly, Maeda et al. [13] reported that dental students achieved a correct response rate of 53.4% when identifying 28 anatomical structures on PRs. Soltanimehr et al. [18] focused on students’ knowledge of jaw lesions; however, their study primarily compared two educational methods rather than assessing real-case diagnostic accuracy. Their evaluation relied on theoretical multiple-choice tests and structured reporting tasks rather than actual diagnostic performance [18]. In our study, the mean accuracy rate for identifying normal anatomical structures was 59.9%, based on 10 questions. However, since our assessment included only half the number of anatomical structures questions compared to previous studies, this numerical similarity should be interpreted with caution and does not necessarily imply equivalent levels of understanding the anatomical structures.

**Table 4:** Summary of previous studies evaluating normal anatomical structures or pathologic conditions on panoramic radiographs

No.	Year	Authors (Nation)	Aim	Methods	Conclusions	Participants
*Normal anatomical structures*						
1	2017	İlgüy et al. [12] (Turkey)	Evaluation of knowledge retention in radiographic interpretation	26 anatomical structures shown in 10 PAs and 5 PRs 19 image acquisition errors shown in PR	Third-year students achieved the highest mean success rate of 59.62%	152 students
2	2018	Maeda et al. [13] (Japan)	Evaluation of students’ understanding of panoramic anatomical landmarks	28 anatomical structures (bone, soft tissue, ghost images)	Soft tissue structures showed significantly lower accuracy	119 students
*Pathological conditions*						
1	2016	Azimi et al. [14] (Iran)	Assessment of diagnostic skills for jaw lesions	Final diagnosis for 10 cases using PRs Descriptive diagnosis for 3 cases using clinical information and radiographs	Mean score: 14.32/20	36 students
2	2019	Soltanimehr et al. [18] (Iran)	Assessment of theoretical knowledge of dental students	40 multiple-choice questions	Mean score range: 14.14–16.60	39 students

*PA* periapical radiograph, *PR* panoramic radiograph

Azimi et al. [14] assessed 36 students using two methods: one based solely on PR, and the other combining PR with accompanying clinical information. They reported an approximate diagnostic accuracy rate of 70% for each method, based on the conversion of test scores into percentage-based accuracy rates. In contrast, we used only an image-based diagnostic method. This approach was adopted to reflect the educational goals of oral radiology training, which emphasize the ability to identify and interpret all lesions visible on PRs. Given that jaw lesions are frequently detected incidentally in asymptomatic patients, it is essential for students to develop the ability to interpret such findings independently of clinical situations.

Compared to their result using the PR image-based method, our diagnostic accuracy rate was lower, not exceeding 50%. This discrepancy can be attributed to differences in case selection, sample size, and grading methods. Azimi et al. [14] used only 10 cases (3 benign tumors, 3 cysts, 3 other bone diseases, and 1 normal bone marrow), whereas our study covered 40 cases, including inflammation and malignancy. And our sample size was larger (125 vs. 36 students), which may have led to greater variability in diagnostic performance. Furthermore, considering the grading method, we allowed only one predetermined diagnosis, whereas Azimi et al. accepted three possible answers as correct.

Our study makes three significant contributions. First, we included a wide range of jaw lesions. Our study covered less common but essential lesions including schwannoma and ossifying fibroma. And, if relatively frequent jaw lesions such as odontogenic keratocysts or ameloblastomas, by utilizing several cases that occurred in the anterior or posterior teeth region, we assessed whether students could recognize location-specific characteristics of these lesions.

Second, our analysis of diagnostic accuracy across the four lesion categories revealed a descending trend, in the following order: cyst, benign tumor, inflammation or malignancy, and other bone lesion. This pattern suggests that lesions with well-defined borders or corticated margin are more easily identified by students than those with ill-defined or infiltrative margins. Both cysts and benign tumors typically demonstrate slow growth and remodeling of adjacent bone, resulting in clear demarcation. This may explain the lack of a statistically significant difference in diagnostic accuracy between these two lesion types. In contrast, inflammation or malignancy tend to present with diffuse bone destruction, which complicates radiographic interpretation. For example, in osteomyelitis, it is often difficult to distinguish sequestrum from normal bone, which may reduce diagnostic accuracy. Similarly, in malignancies involving the maxillary sinus wall and palatine bone, overlapping anatomical structures and students’ lack of anatomical knowledge make diagnosis even more difficult. Of note, the diagnostic accuracy for other bone lesion was the lowest, at 32.7%. These lesions often present mixed radiographic features, which may change with maturation and alterations in trabecular structure. The students’ limited exposure to such diverse and atypical radiologic presentations likely contributed to the marked decrease in diagnostic accuracy for this category.

Third, by dividing the 125 students into three groups based on their anatomical structure scores and comparing their diagnostic accuracy for jaw lesions, we observed statistically significant differences among the groups. Students in the highest-scoring group demonstrated a significantly greater accuracy rate in diagnosing jaw lesions, suggesting that robust knowledge of anatomical structures is indispensable for accurate interpretation of PR.

Despite these contributions, several limitations must be acknowledged. First, this study involved dental students from a single university in Korea. It showed limited statistical power (0.64), acceptable only for a preliminary study. A larger cohort study including participants from various countries and academic backgrounds is needed. And although this study controlled student-related variables including gender and academic year, the potential impact of these factors on diagnostic performance warrants further investigation. In particular, the potential confounding effect of students’ baseline academic performance (e.g., coursework score) was not addressed. Future studies should consider controlling for baseline scientific proficiency to better clarify independent associations. Second, the diagnostic tasks were based solely on radiographic images of typical and representative cases without accompanying clinical information, which aligns with the educational purpose of evaluating interpretive skills under standardized conditions. However, this design may limit the generalizability of the findings to real-world clinical settings, where atypical presentations can also occur. Lastly, qualitative approaches, including surveys on diagnostic challenges and focus-group interviews on instructional effectiveness, may provide deeper pedagogical insights. A mixed-methods design that integrates quantitative and qualitative analyses could lead to a more comprehensive understanding of how to improve radiographic anatomy education.

Therefore, we suggest that anatomy education in PR should be reinforced. To effectively teach anatomical structures, practical training methods that utilize advanced technologies are necessary. Recent studies have reported that virtual and interactive digital technologies in dental education foster greater student motivation and satisfaction. Furthermore, these approaches enhanced spatial understanding and diagnostic accuracy in radiographic interpretation [18–21]. Additionally, a scaffolded educational approach—wherein students first learn to identify ideal anatomic landmarks and then progress to recognizing these structures when they are blurred, distorted, or overlapping with other structures—can be considered. An adaptive learning framework supported by artificial intelligence (AI), tailored to individual proficiency levels, could better accommodate diverse student needs. For instance, generative adversarial networks can create PRs simulating pathological changes, enriching curricula with diverse clinical scenarios [22]. Integrating AI-generated radiographic data is expected to enhance students’ diagnostic skills and overall competency in radiographic interpretation. Future studies should consider evaluating the effectiveness of these methodological.

## Conclusion

This study offers valuable pedagogical insights by empirically highlighting the essential role of dental students’ proficiency in recognizing normal anatomical structures for the accurate diagnosis of jaw lesions. Moreover, it emphasizes the importance of educational approaches that strengthen students’ understanding of normal anatomy as visualized on PR.

## Supplementary Information

Supplementary Material 1.

## Data Availability

Data is provided within the supplementary information files.

## Abbreviations

PR: Panoramic radiograph
AI: Artificial intelligence
