# Supplementary material for: Assessing dental students’ knowledge of panoramic radiographs and the importance of normal anatomy education
Source: BMC Med Educ. 2025 Aug 30;25:1235. doi: 10.1186/s12909-025-07829-w (PMC12398122; doi:10.1186/s12909-025-07829-w)
Supplement: Supplementary file 1 — Supplementary Material 1. [file 12909_2025_7829_MOESM1_ESM.pdf]

Slide test

Date : 2023-11-08

1. Write down the name of the anatomical structure pointed to by the yellow arrow.

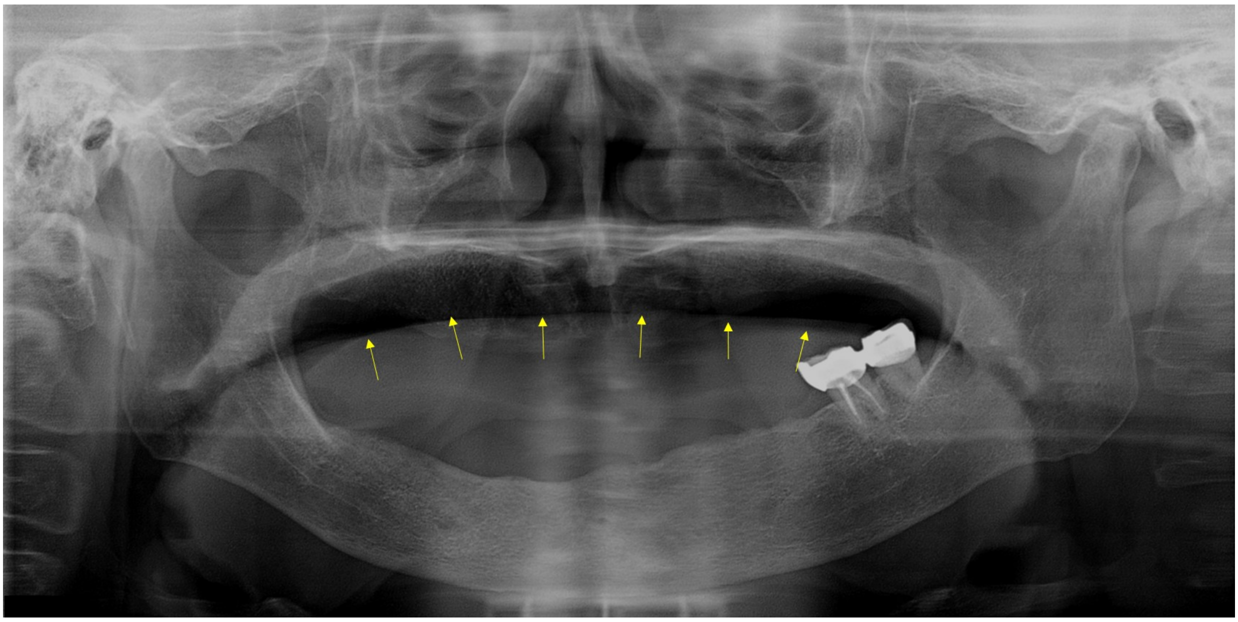

dorsal surface of tongue

Slide test

Date : 2023-11-08

2. Write down the name of the anatomical structure pointed to by the yellow arrow.

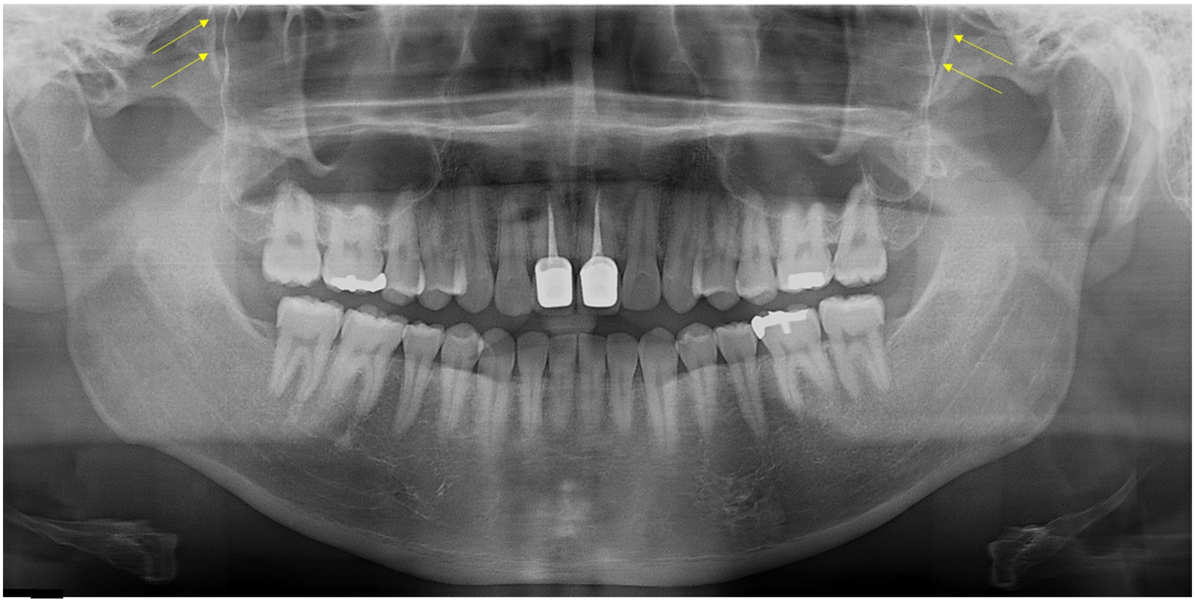

pterygomaxillary fissure

Slide test

Date : 2023-11-08

3. Write down the name of the anatomical structure pointed to by the yellow arrow.

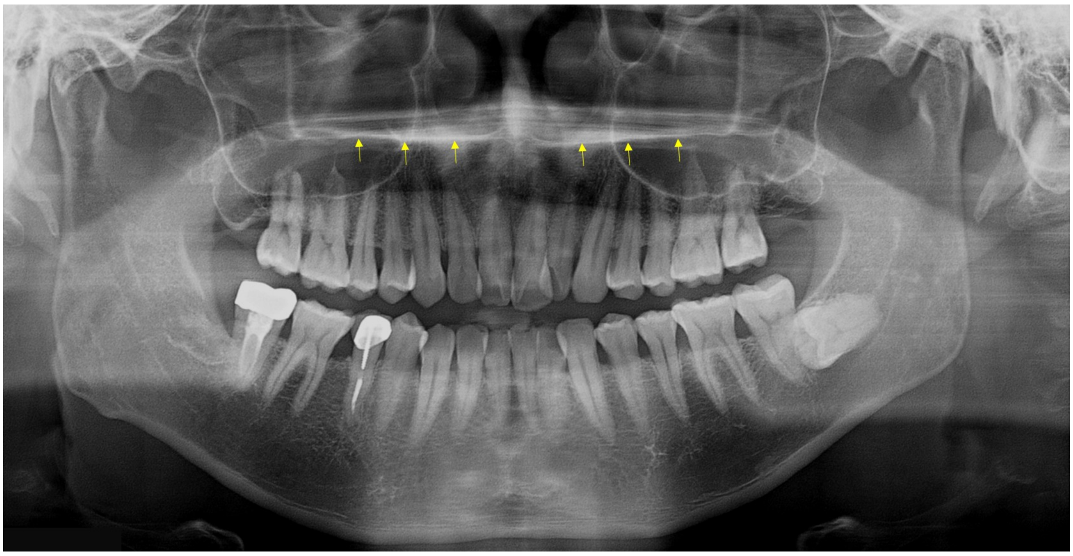

hard palate

Slide test

Date : 2023-11-08

4. Write down the name of the anatomical structure pointed to by the yellow arrow.

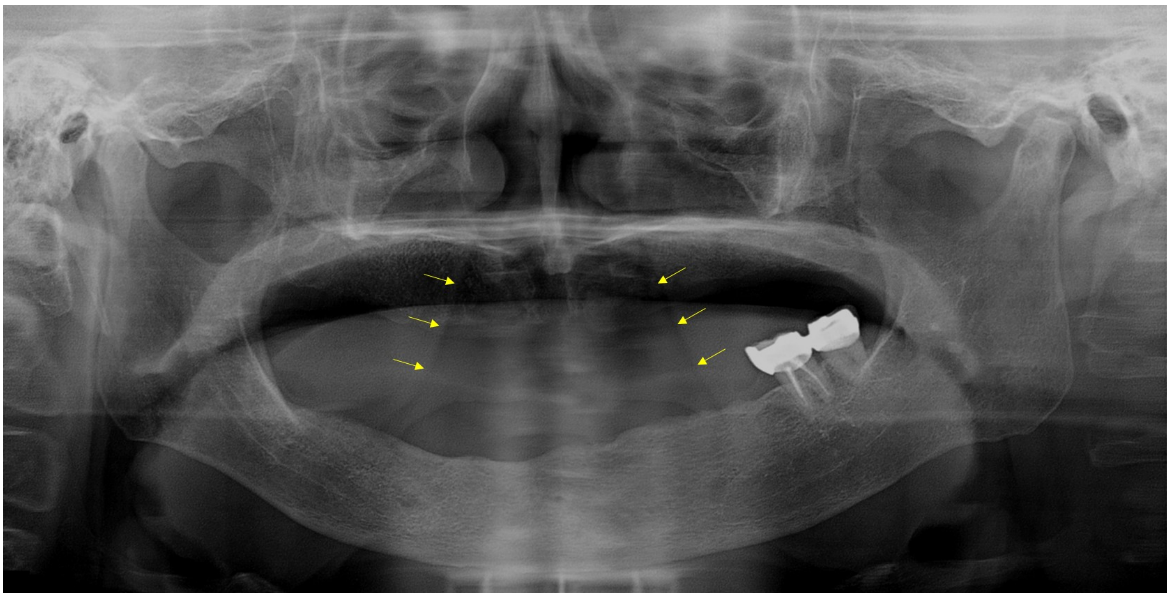

nasolabial fold

Slide test

Date : 2023-11-08

5. Write down the name of the anatomical structure pointed to by the yellow arrow.

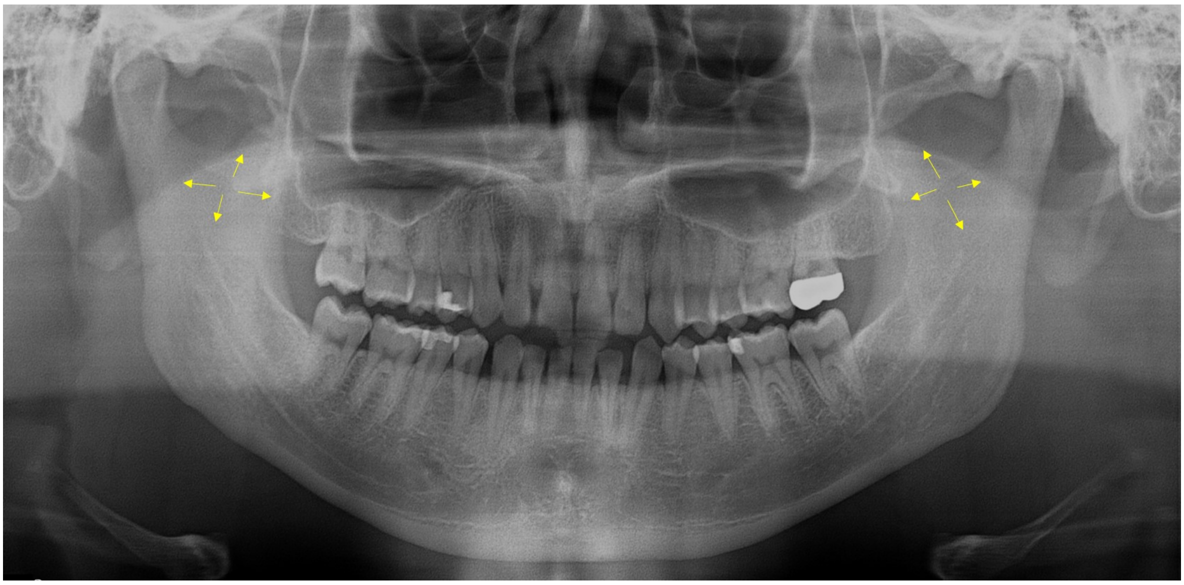

soft palate

Slide test

Date : 2023-11-08

6. Write down the name of the anatomical structure pointed to by the yellow arrow.

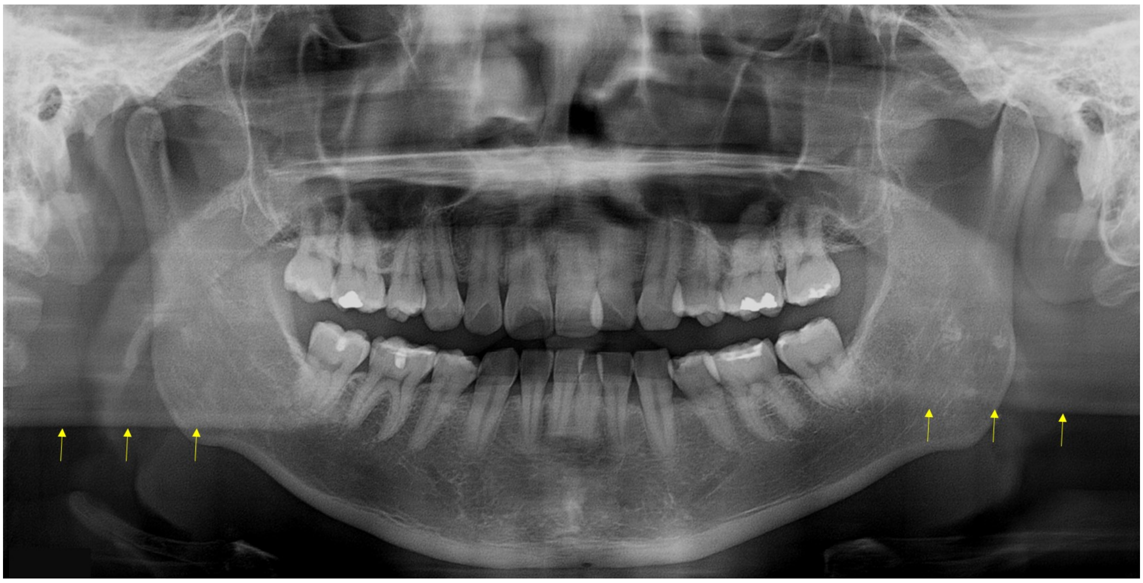

ghost image of mandible angle

Slide test

Date : 2023-11-08

7. Write down the name of the anatomical structure pointed to by the yellow arrow.

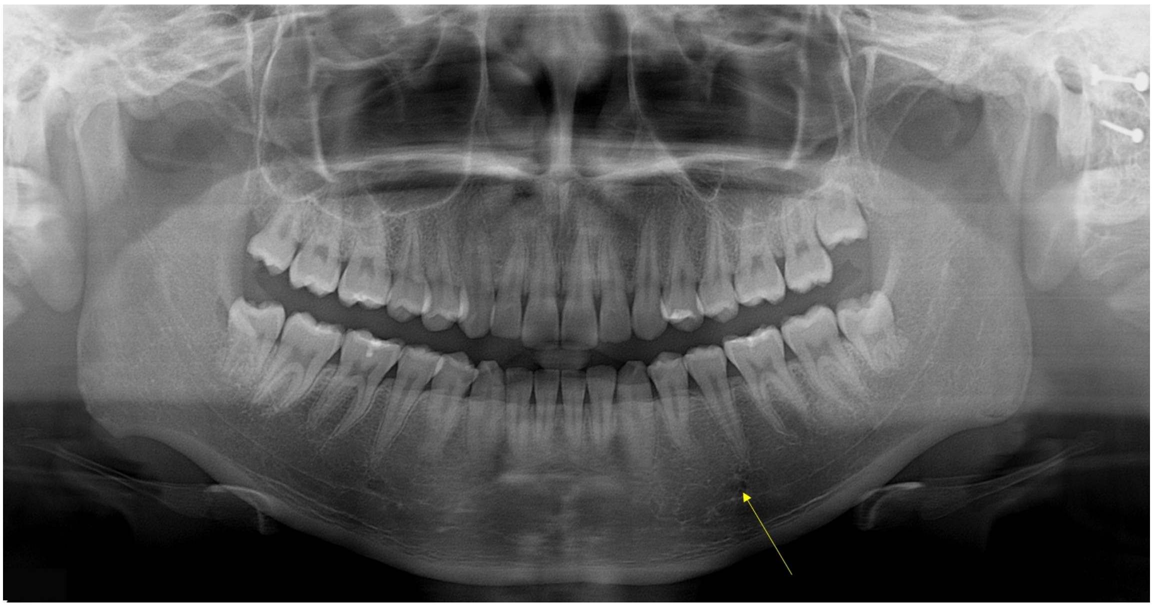

mental foramen

Slide test

Date : 2023-11-08

8. Write down the name of the anatomical structure pointed to by the yellow arrow.

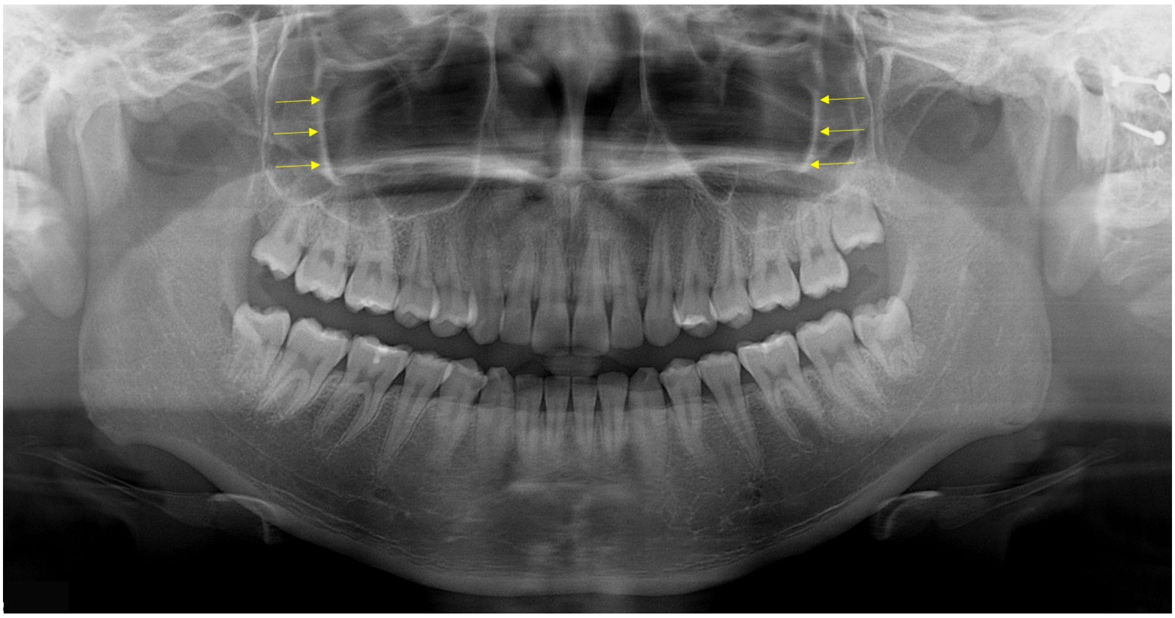

innominate line

Slide test

Date : 2023-11-08

9. Write down the name of the anatomical structure pointed to by the yellow arrow.

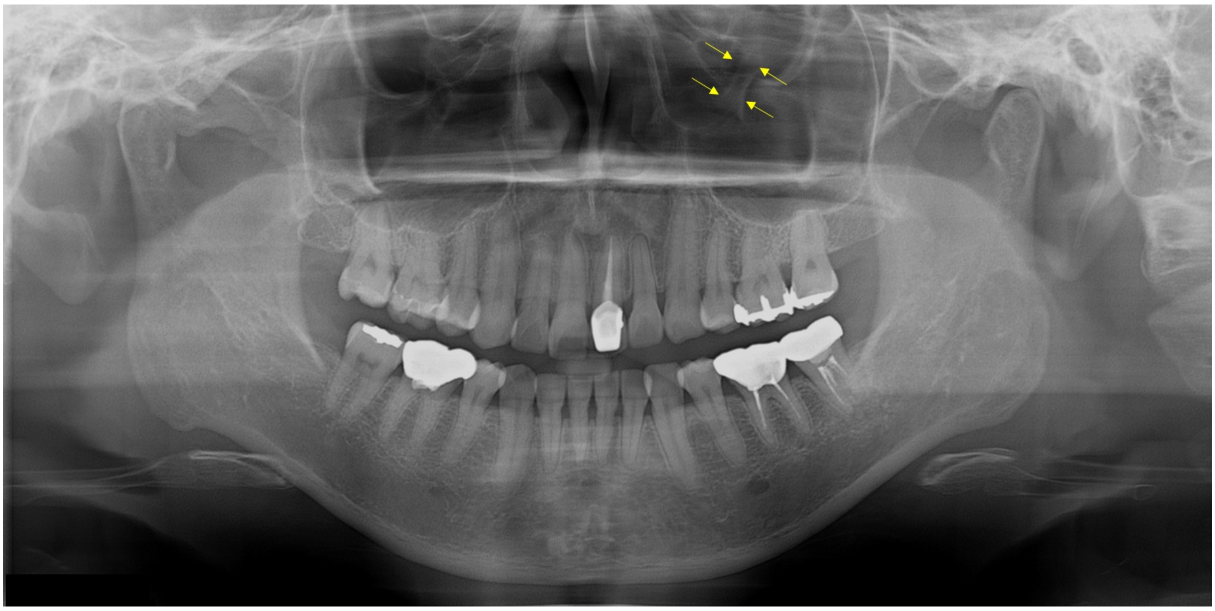

infraorbital canal

Slide test

Date : 2023-11-08

10. Write down the name of the anatomical structure pointed to by the yellow arrow.

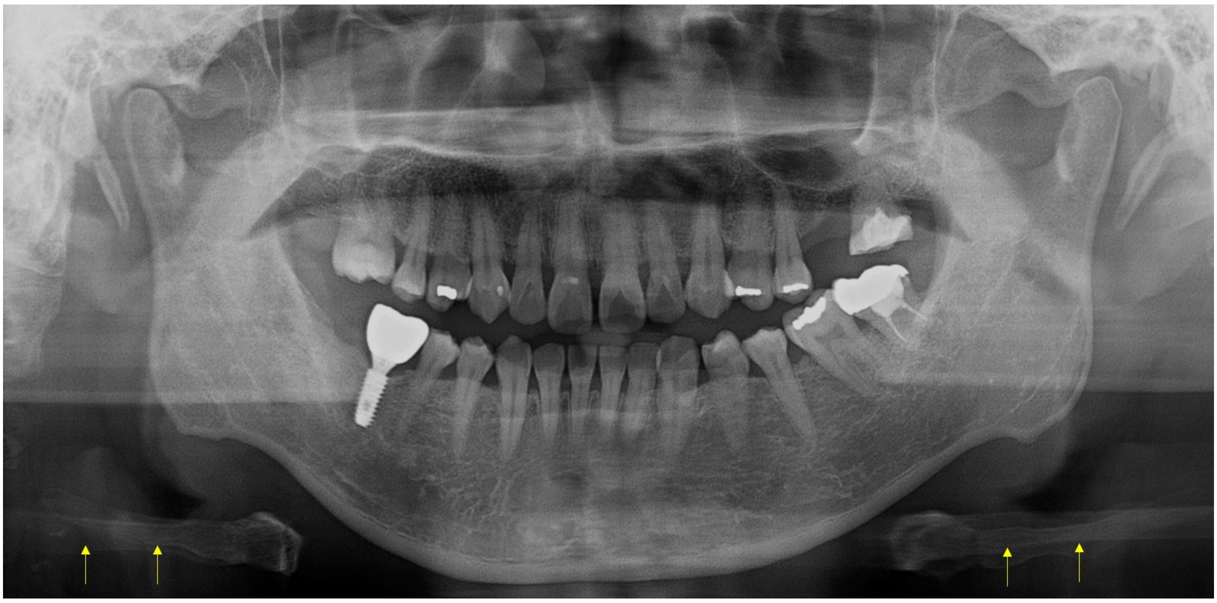

hyoid bone

Slide test

Date: 2023-11-08

11. Choose the number according to the lesion location observed in the following panorama and write down its diagnosis.

1) Right maxilla
2) Anterior maxilla
3) Left maxilla
4) Right mandible
5) Anterior mandible
6) Left mandible

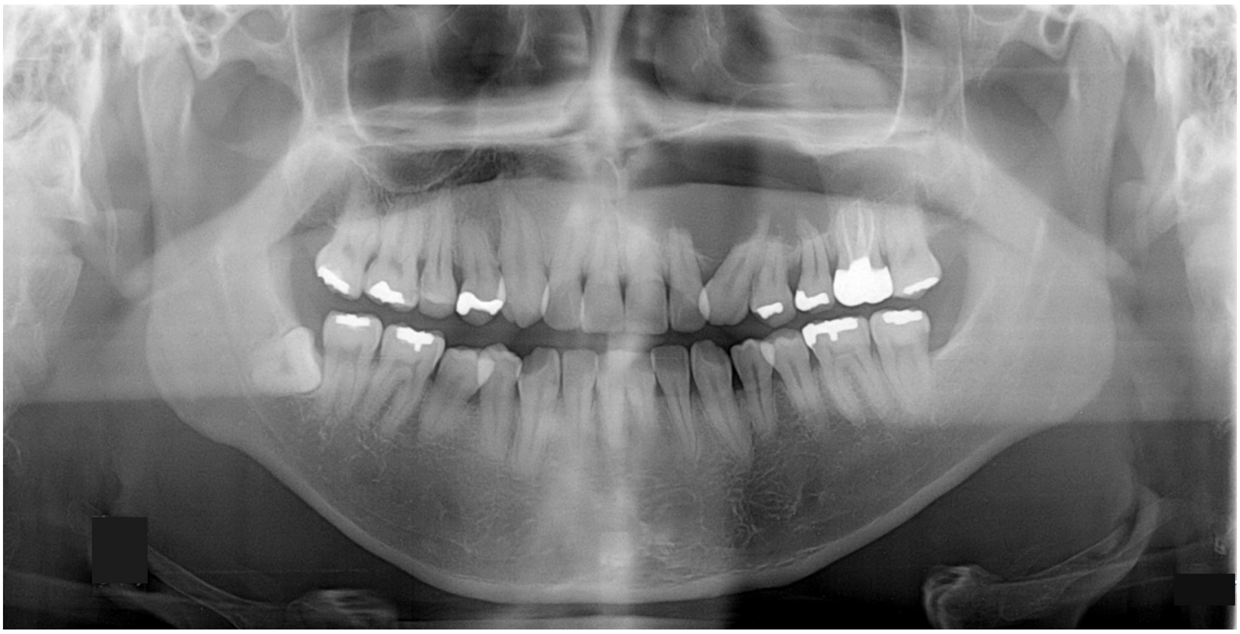

3-ameloblastoma

12. Choose the number according to the lesion location observed in the following panorama and write down its diagnosis.

1) Right maxilla
2) Anterior maxilla
3) Left maxilla
4) Right mandible
5) Anterior mandible
6) Left mandible

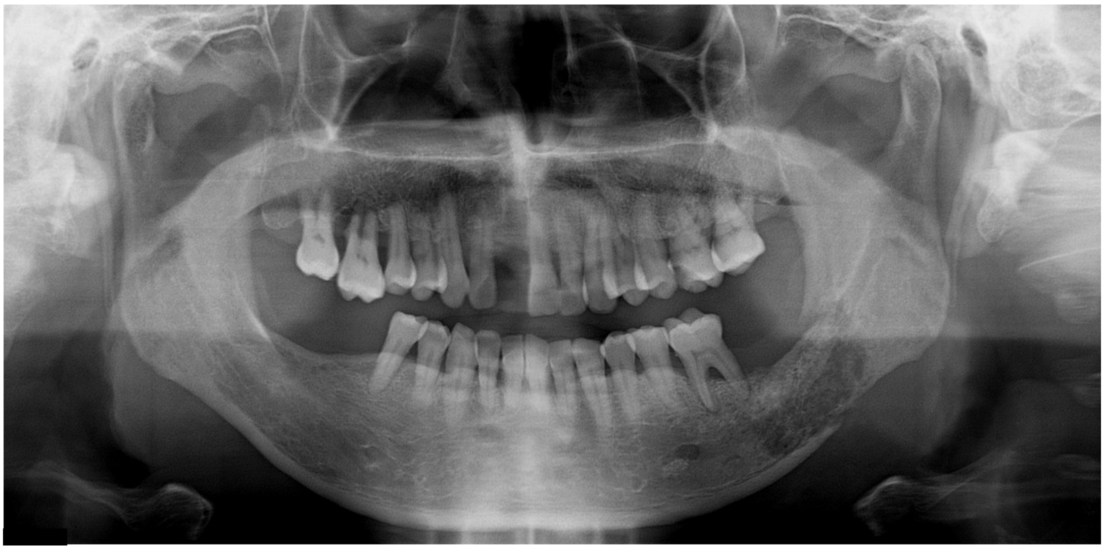

6-osteomyelitis

Slide test

Date: 2023-11-08

13. Choose the number according to the lesion location observed in the following panorama and write down its diagnosis.

1) Right maxilla
2) Anterior maxilla
3) Left maxilla
4) Right mandible
5) Anterior mandible
6) Left mandible

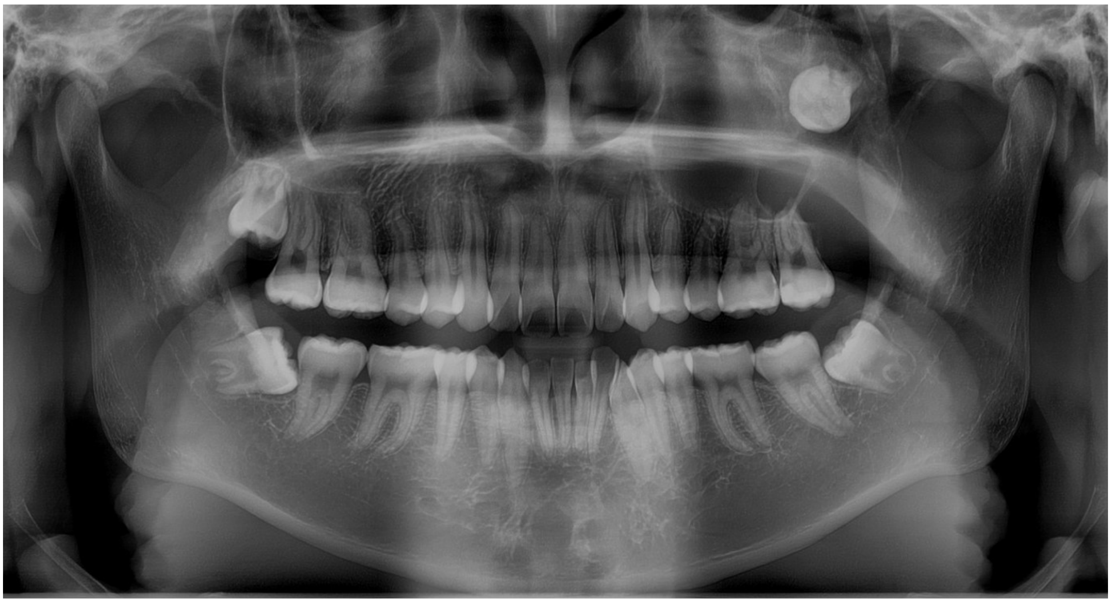

3-odontogenic keratocyst

Slide test

Date: 2023-11-08

14. Choose the number according to the lesion location observed in the following panorama and write down its diagnosis.

1) Right maxilla
2) Anterior maxilla
3) Left maxilla
4) Right mandible
5) Anterior mandible
6) Left mandible

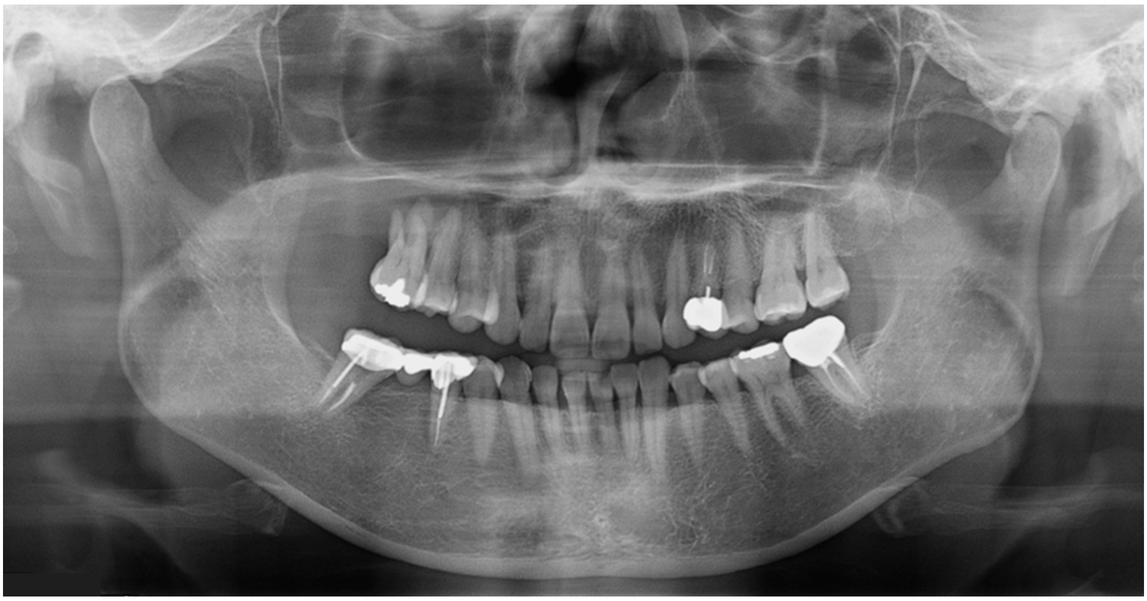

1-malignancy

Slide test

Date: 2023-11-08

15. Choose the number according to the lesion location observed in the following panorama and write down its diagnosis.

1) Right maxilla
2) Anterior maxilla
3) Left maxilla
4) Right mandible
5) Anterior mandible
6) Left mandible

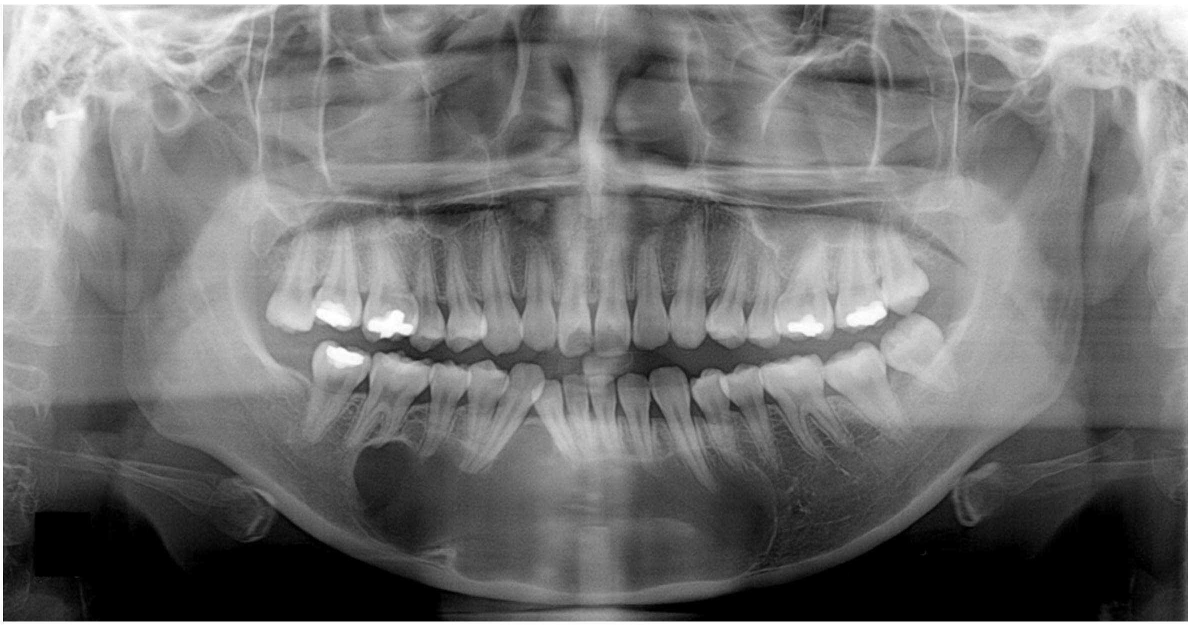

5-odontogenic keratocyst

Slide test

Date: 2023-11-08

16. Choose the number according to the lesion location observed in the following panorama and write down its diagnosis.

1) Right maxilla
2) Anterior maxilla
3) Left maxilla
4) Right mandible
5) Anterior mandible
6) Left mandible

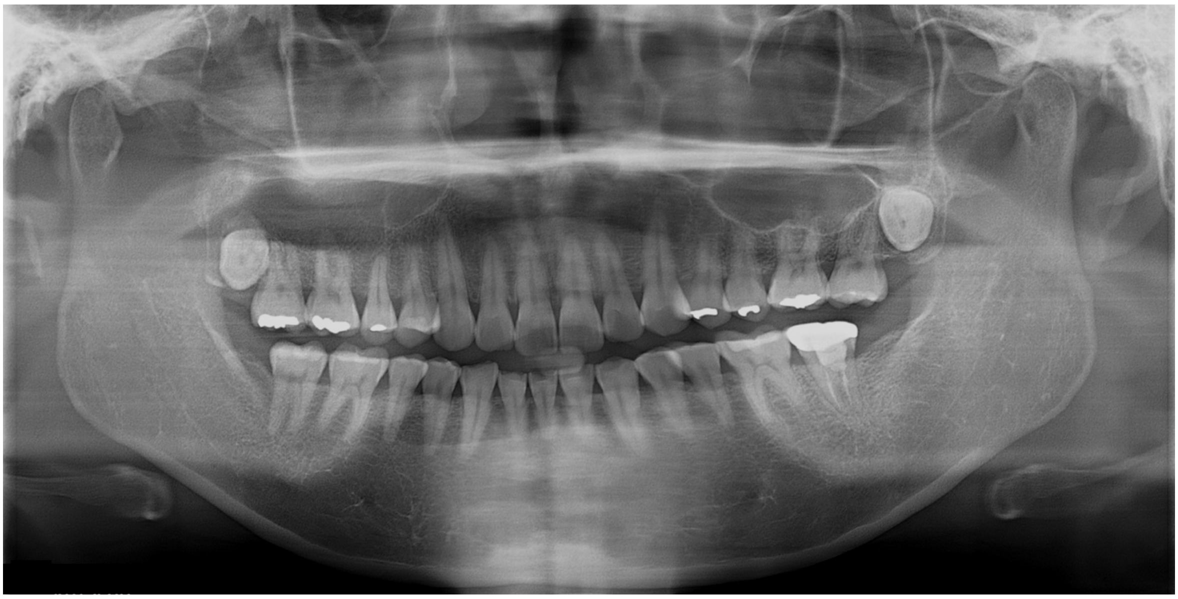

1-malignancy

Slide test

Date: 2023-11-08

17. Choose the number according to the lesion location observed in the following panorama and write down its diagnosis.

1) Right maxilla
2) Anterior maxilla
3) Left maxilla
4) Right mandible
5) Anterior mandible
6) Left mandible

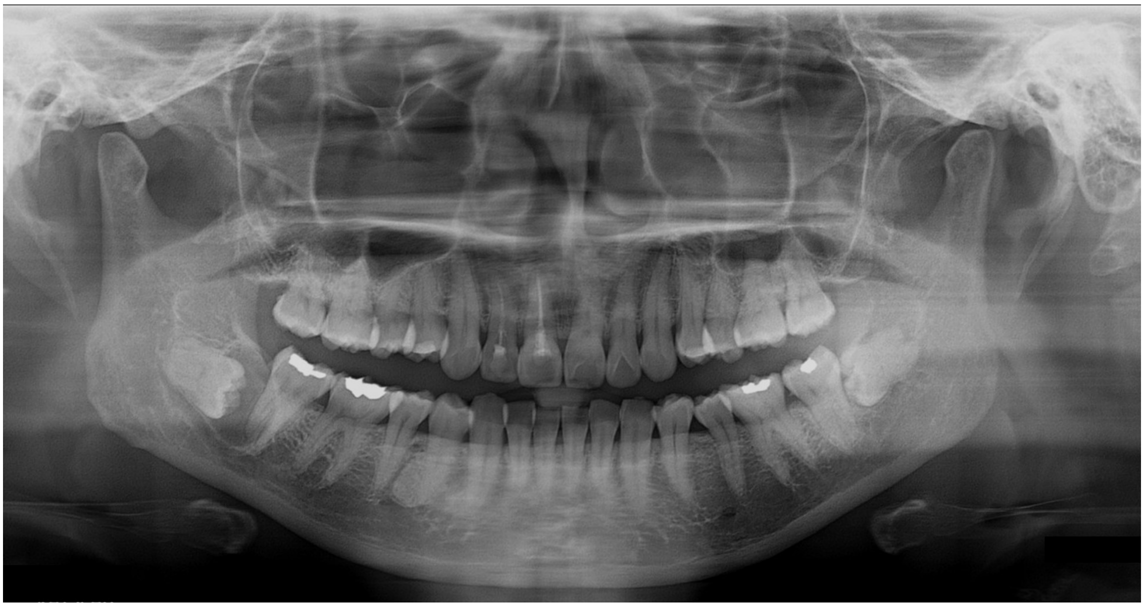

4-dentigerous cyst

Slide test Date: 2023-11-08

18. Choose the number according to the lesion location observed in the following panorama and write down its diagnosis.

1) Right maxilla
2) Anterior maxilla
3) Left maxilla
4) Right mandible
5) Anterior mandible
6) Left mandible

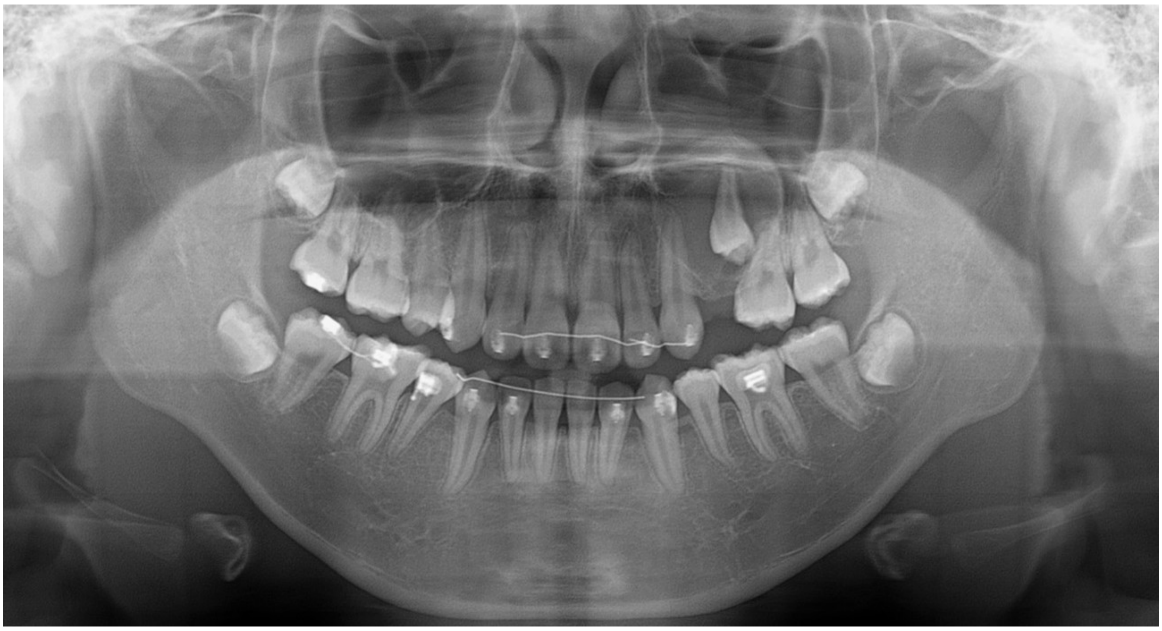

3-adenoid odontogenic tumor

Slide test

Date: 2023-11-08

19. Choose the number according to the lesion location observed in the following panorama and write down its diagnosis.

1) Right maxilla
2) Anterior maxilla
3) Left maxilla
4) Right mandible
5) Anterior mandible
6) Left mandible

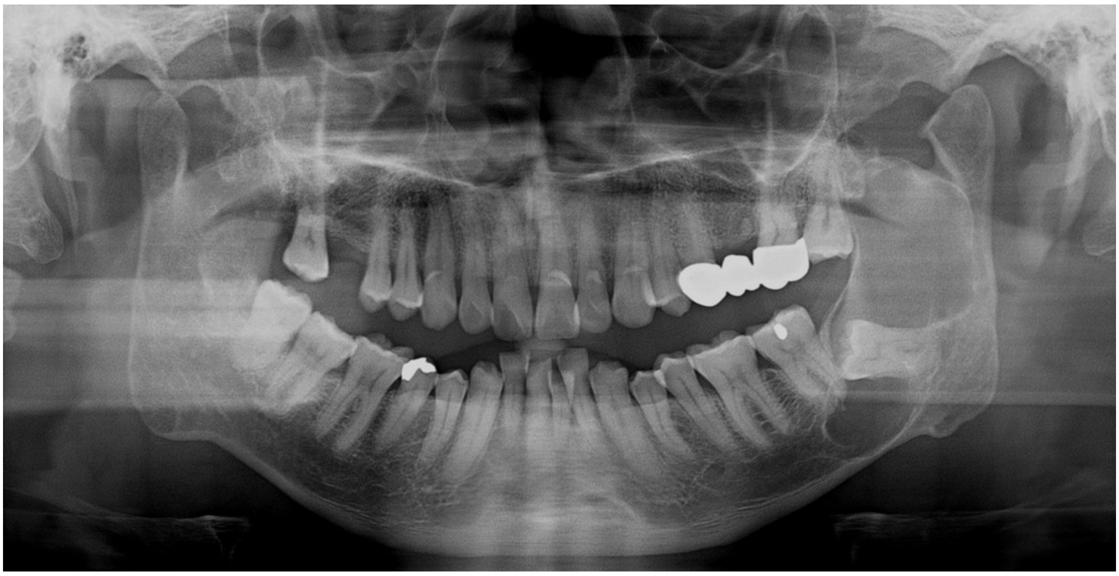

6-odontogenic keratocyst

Slide test

Date: 2023-11-08

20. Choose the number according to the lesion location observed in the following panorama and write down its diagnosis.

1) Right maxilla
2) Anterior maxilla
3) Left maxilla
4) Right mandible
5) Anterior mandible
6) Left mandible

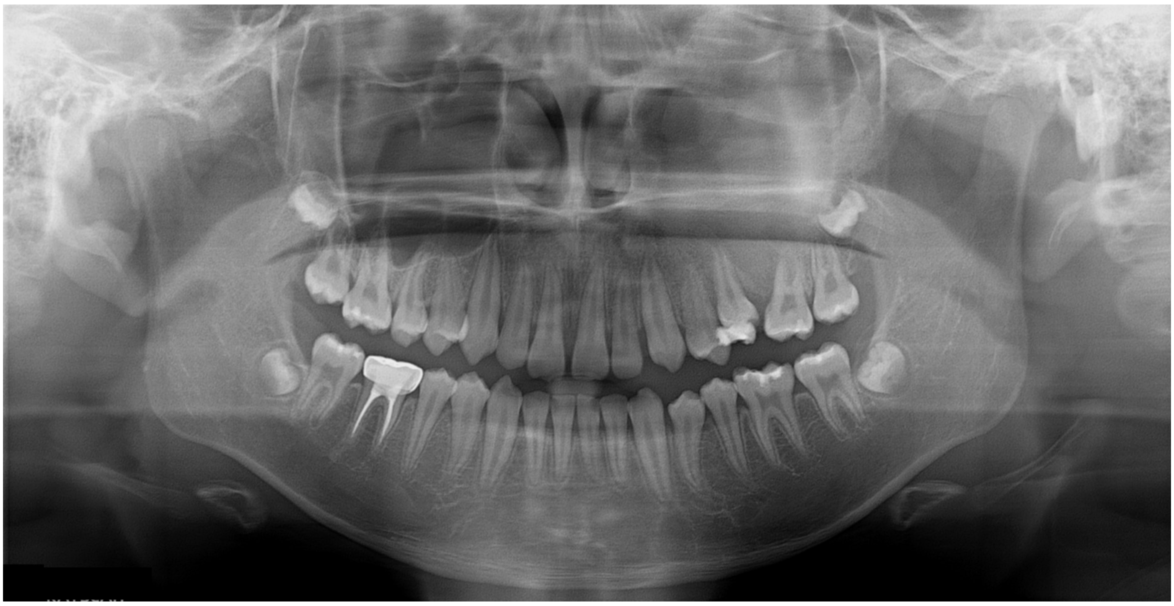

3-fibrous dysplasia

Slide test

Date: 2023-11-08

21. Choose the number according to the lesion location observed in the following panorama and write down its diagnosis.

1) Right maxilla
2) Anterior maxilla
3) Left maxilla
4) Right mandible
5) Anterior mandible
6) Left mandible

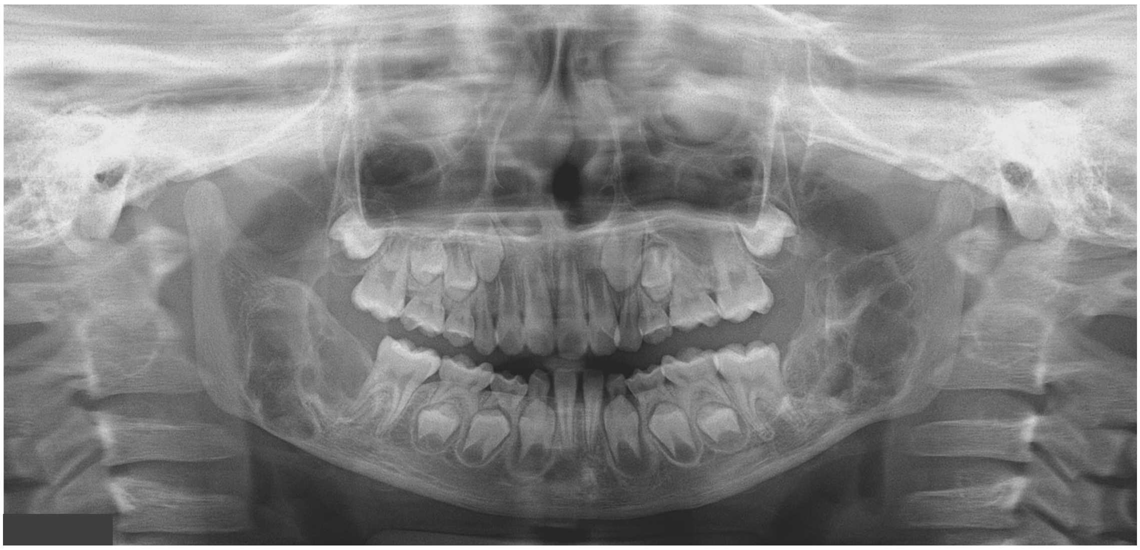

4,6-cherubism

Slide test

Date: 2023-11-08

22. Choose the number according to the lesion location observed in the following panorama and write down its diagnosis.

1) Right maxilla
2) Anterior maxilla
3) Left maxilla
4) Right mandible
5) Anterior mandible
6) Left mandible

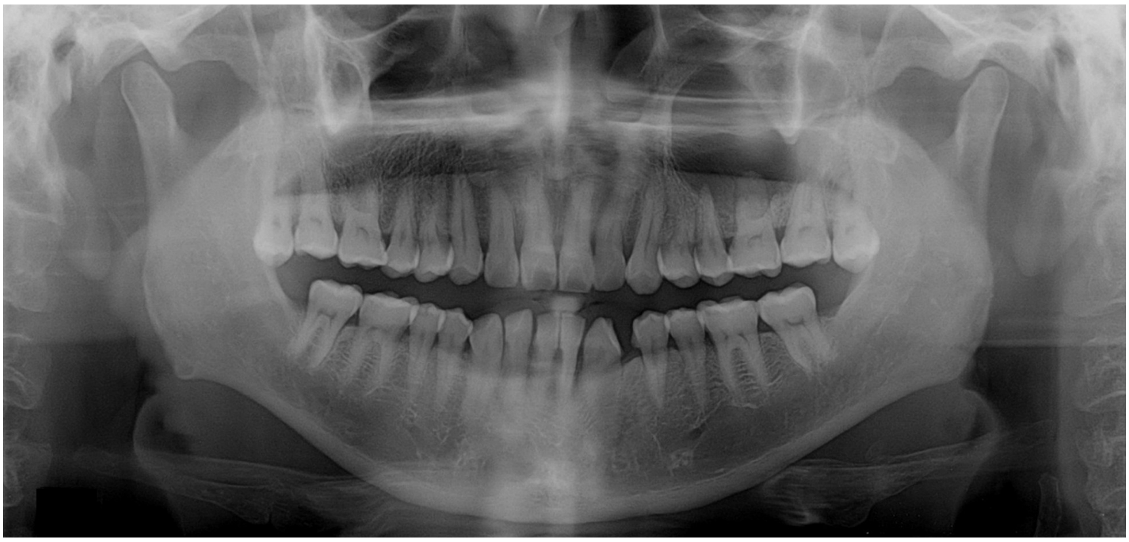

3-post operative maxillary cyst

Slide test Date: 2023-11-08

23. Choose the number according to the lesion location observed in the following panorama and write down its diagnosis.

1) Right maxilla
2) Anterior maxilla
3) Left maxilla
4) Right mandible
5) Anterior mandible
6) Left mandible

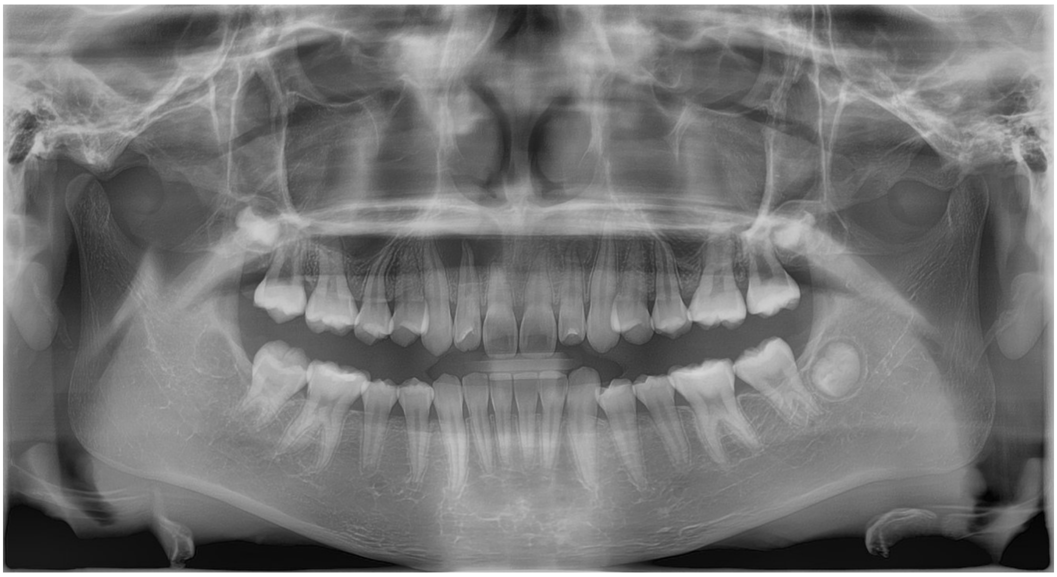

1-radicular cyst

Slide test

Date: 2023-11-08

24. Choose the number according to the lesion location observed in the following panorama and write down its diagnosis.

1) Right maxilla
2) Anterior maxilla
3) Left maxilla
4) Right mandible
5) Anterior mandible
6) Left mandible

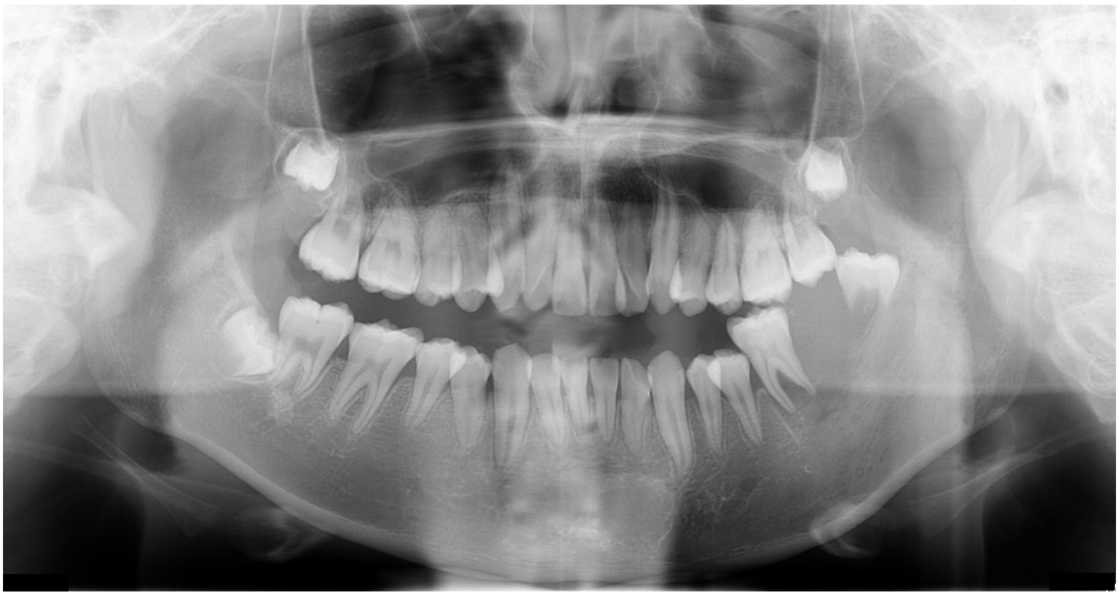

6-malignancy

Slide test

Date: 2023-11-08

25. Choose the number according to the lesion location observed in the following panorama and write down its diagnosis.

1) Right maxilla
2) Anterior maxilla
3) Left maxilla
4) Right mandible
5) Anterior mandible
6) Left mandible

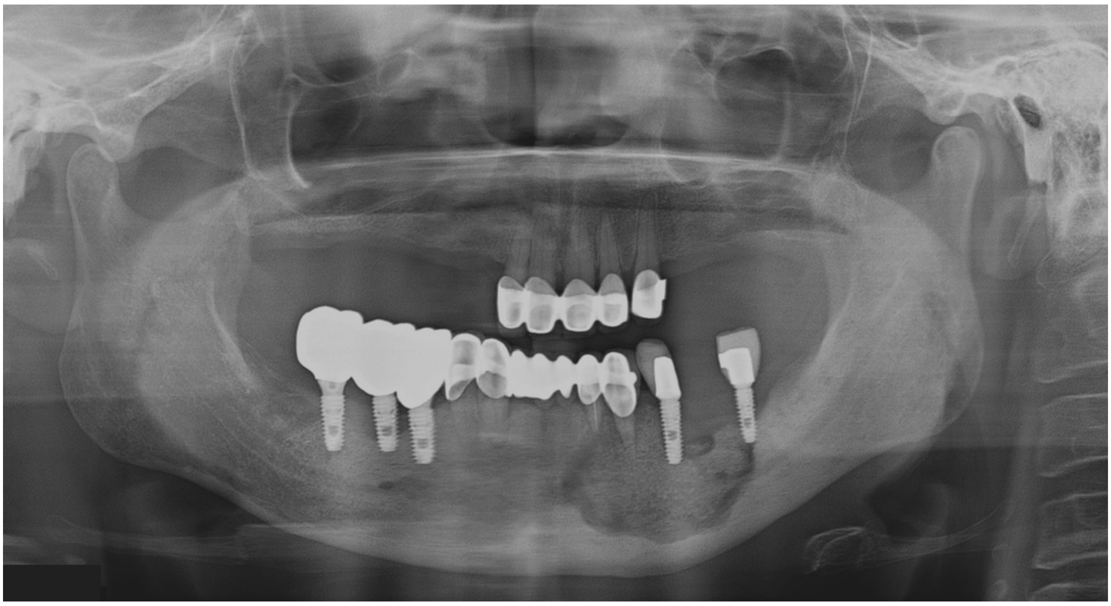

6-osteomyelitis

Slide test

Date: 2023-11-08

26. Choose the number according to the lesion location observed in the following panorama and write down its diagnosis.

1) Right maxilla
2) Anterior maxilla
3) Left maxilla
4) Right mandible
5) Anterior mandible
6) Left mandible

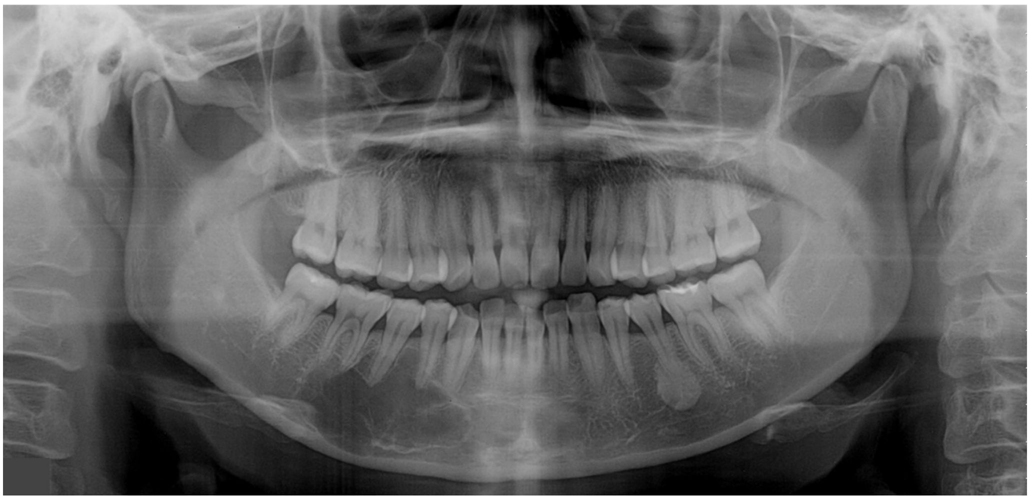

4-odontogenic myxoma

Slide test Date: 2023-11-08

27. Choose the number according to the lesion location observed in the following panorama and write down its diagnosis.

1) Right maxilla
2) Anterior maxilla
3) Left maxilla
4) Right mandible
5) Anterior mandible
6) Left mandible

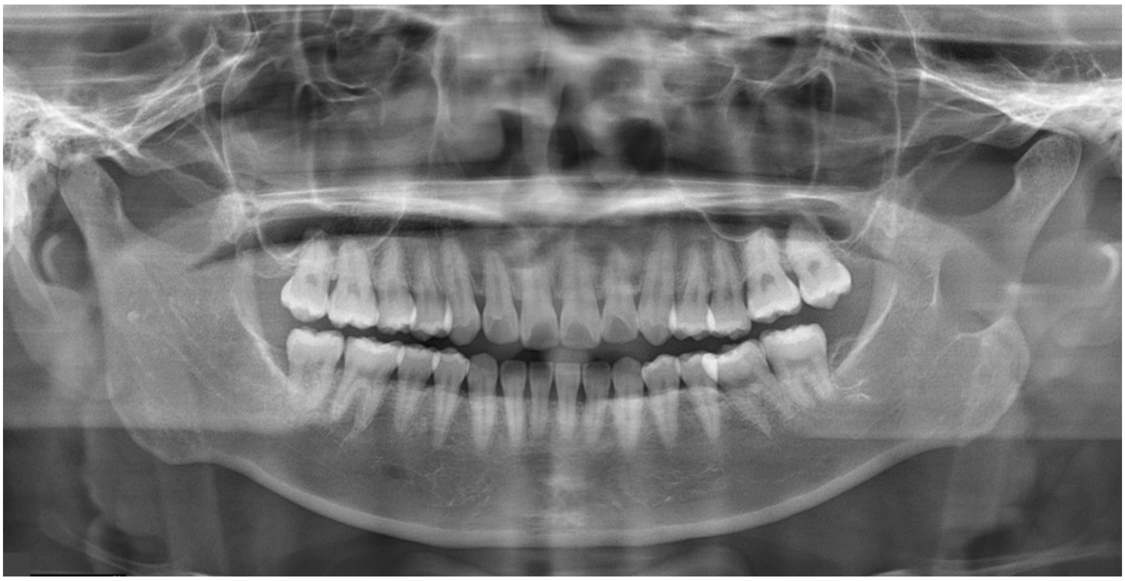

6-schwannoma or 6-neurilemoma

Slide test

Date: 2023-11-08

28. Choose the number according to the lesion location observed in the following panorama and write down its diagnosis.

1) Right maxilla
2) Anterior maxilla
3) Left maxilla
4) Right mandible
5) Anterior mandible
6) Left mandible

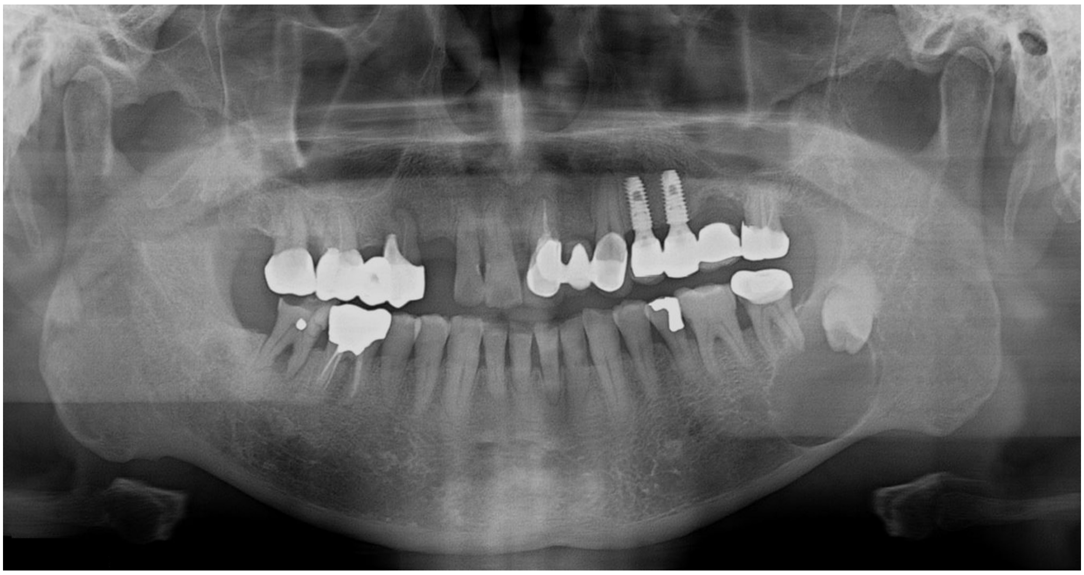

6-dentigerous cyst

Slide test

Date: 2023-11-08

29. Choose the number according to the lesion location observed in the following panorama and write down its diagnosis.

1) Right maxilla
2) Anterior maxilla
3) Left maxilla
4) Right mandible
5) Anterior mandible
6) Left mandible

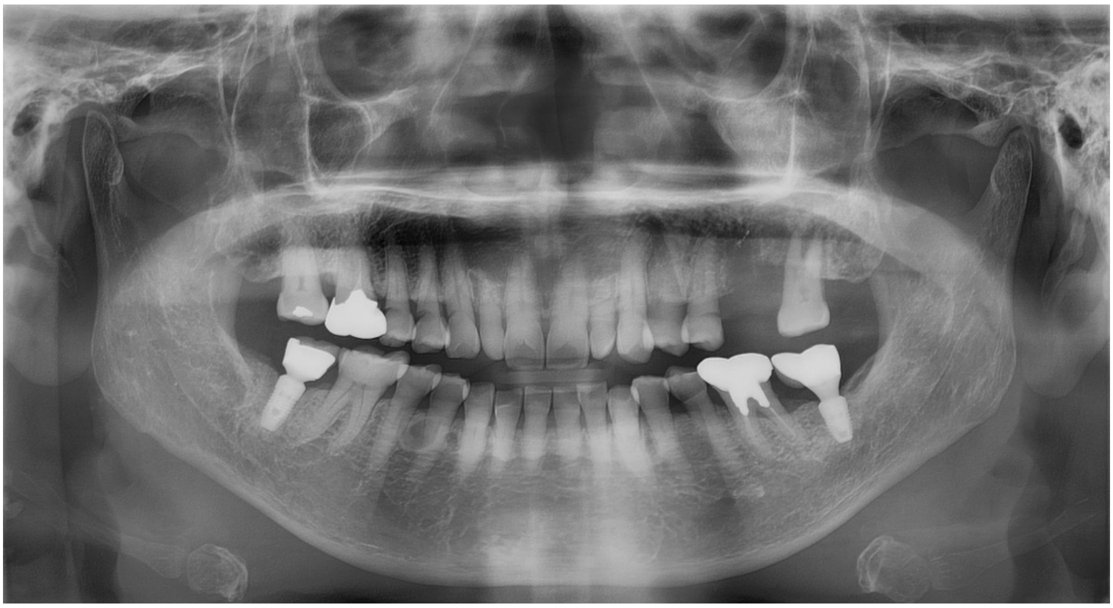

3-osteomyelitis

Slide test

Date: 2023-11-08

30. Choose the number according to the lesion location observed in the following panorama and write down its diagnosis.

1) Right maxilla
2) Anterior maxilla
3) Left maxilla
4) Right mandible
5) Anterior mandible
6) Left mandible

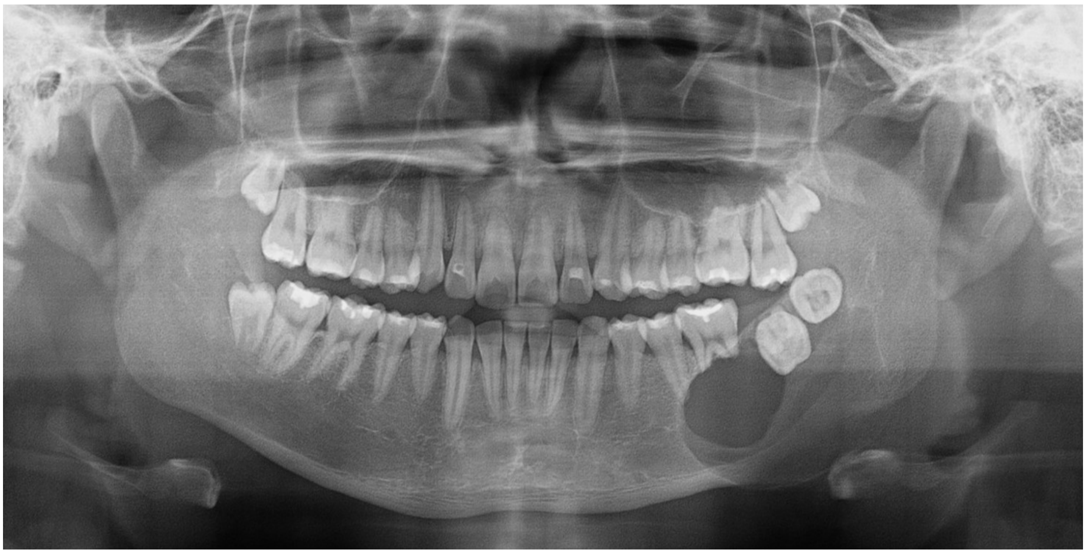

6-ameloblastoma

Slide test

Date: 2023-11-08

31. Choose the number according to the lesion location observed in the following panorama and write down its diagnosis.

1) Right maxilla
2) Anterior maxilla
3) Left maxilla
4) Right mandible
5) Anterior mandible
6) Left mandible

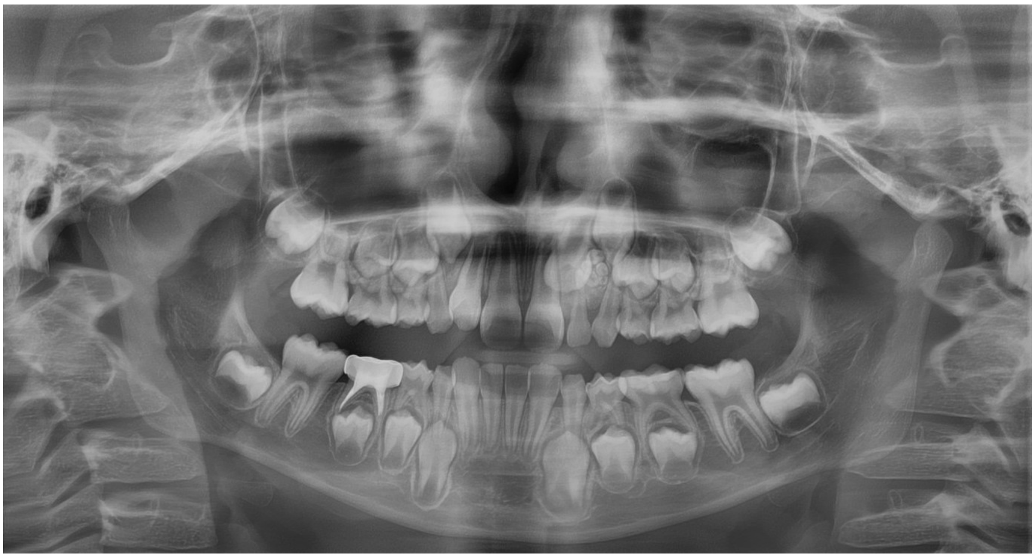

3-odontoma

Slide test

Date: 2023-11-08

32. Choose the number according to the lesion location observed in the following panorama and write down its diagnosis.

1) Right maxilla
2) Anterior maxilla
3) Left maxilla
4) Right mandible
5) Anterior mandible
6) Left mandible

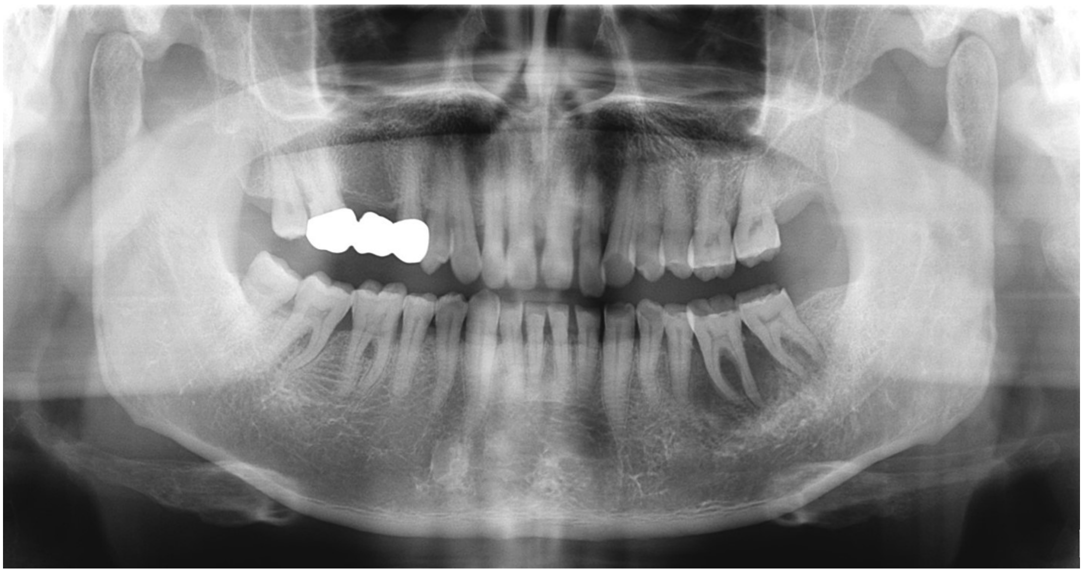

6-malignancy

Slide test

Date: 2023-11-08

33. Choose the number according to the lesion location observed in the following panorama and write down its diagnosis.

1) Right maxilla
2) Anterior maxilla
3) Left maxilla
4) Right mandible
5) Anterior mandible
6) Left mandible

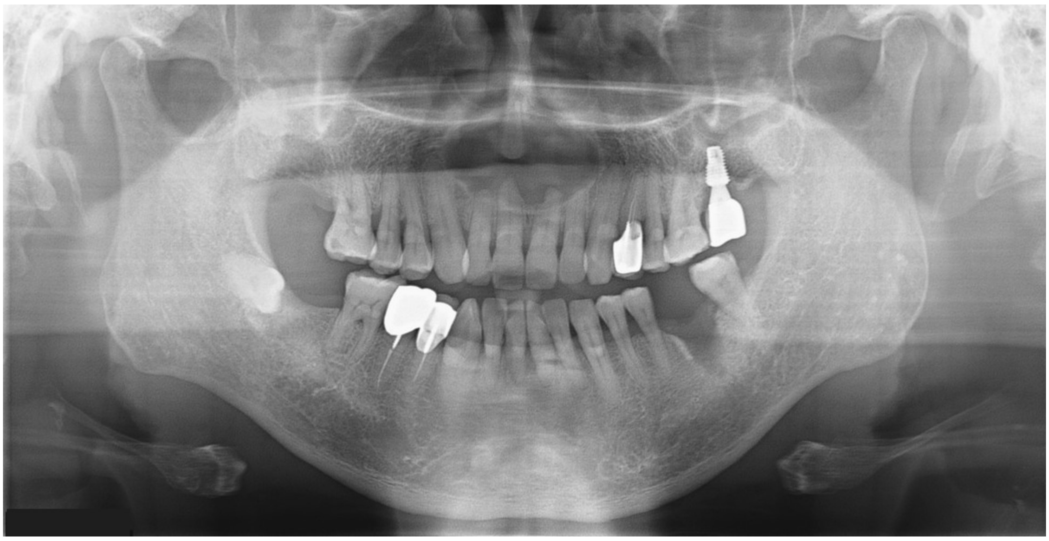

2-nasopalatine canal cyst

Slide test

Date: 2023-11-08

34. Choose the number according to the lesion location observed in the following panorama and write down its diagnosis.

1) Right maxilla
2) Anterior maxilla
3) Left maxilla
4) Right mandible
5) Anterior mandible
6) Left mandible

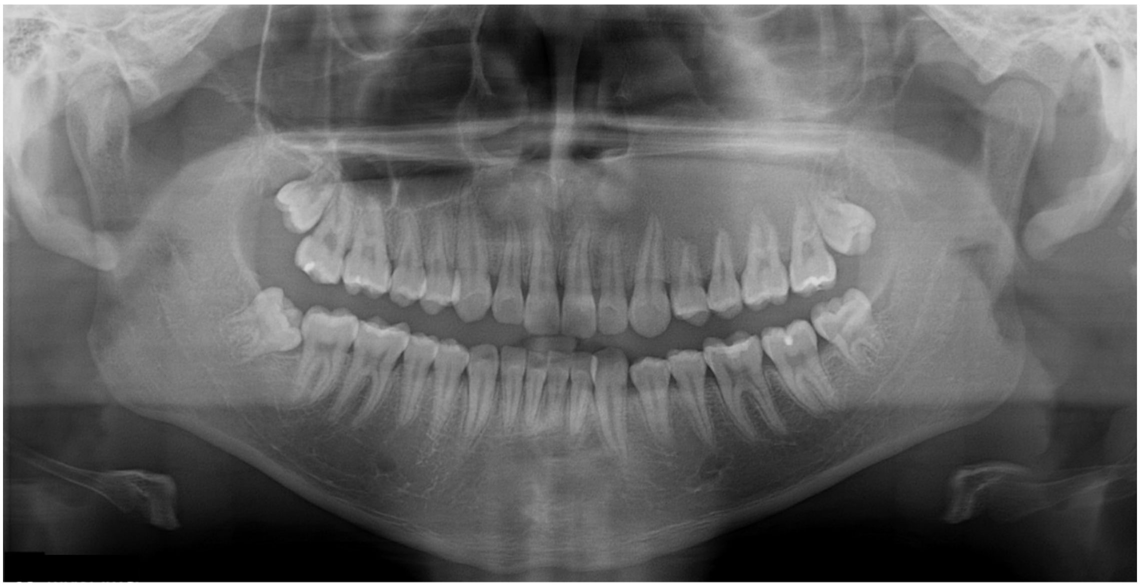

3-fibrous dysplasia

Slide test

Date: 2023-11-08

35. Choose the number according to the lesion location observed in the following panorama and write down its diagnosis.

1) Right maxilla
2) Anterior maxilla
3) Left maxilla
4) Right mandible
5) Anterior mandible
6) Left mandible

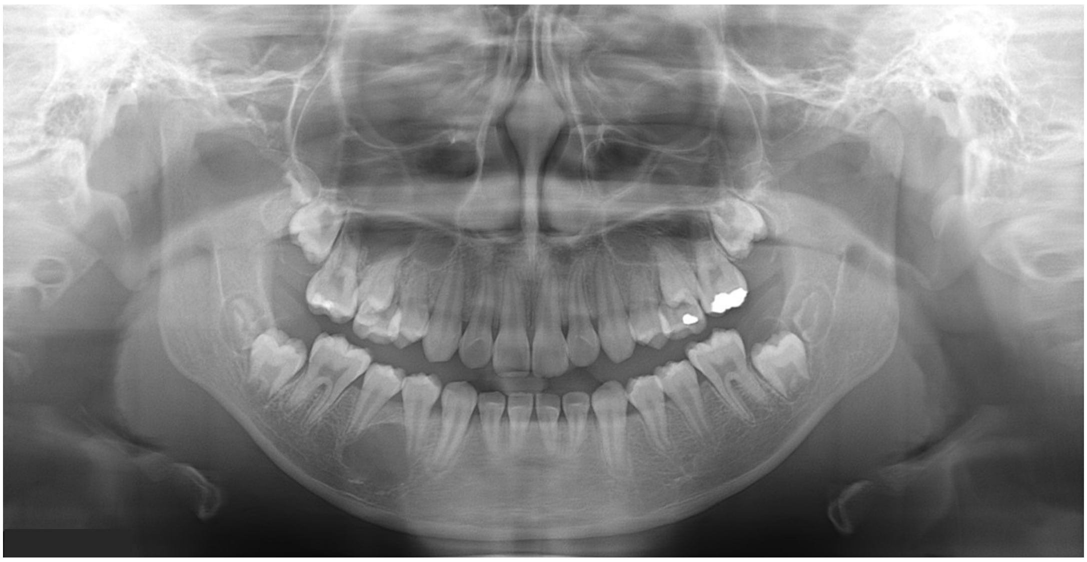

4-simple bone cyst

Slide test

Date: 2023-11-08

36. Choose the number according to the lesion location observed in the following panorama and write down its diagnosis.

1) Right maxilla
2) Anterior maxilla
3) Left maxilla
4) Right mandible
5) Anterior mandible
6) Left mandible

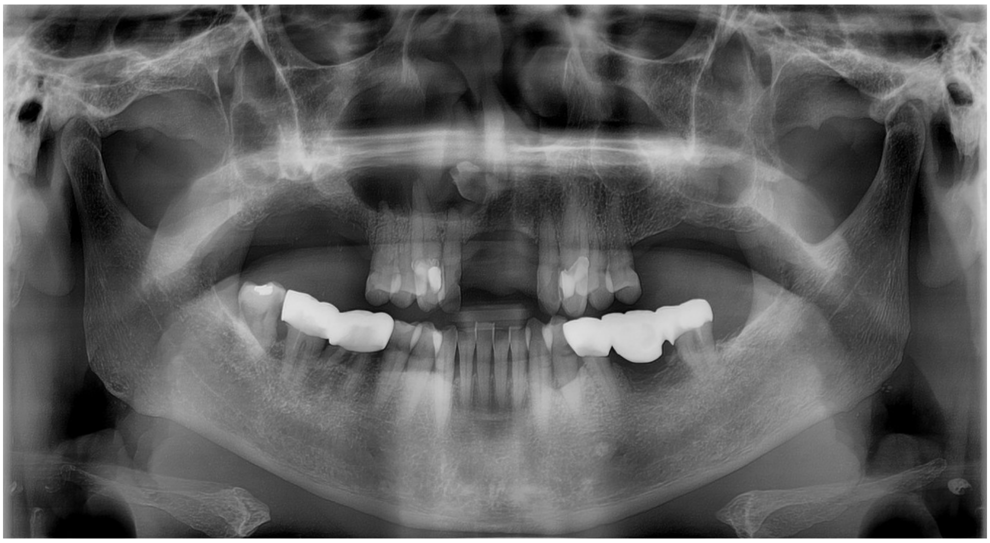

1-dentigerous cyst

Slide test

Date: 2023-11-08

37. Choose the number according to the lesion location observed in the following panorama and write down its diagnosis.

1) Right maxilla
2) Anterior maxilla
3) Left maxilla
4) Right mandible
5) Anterior mandible
6) Left mandible

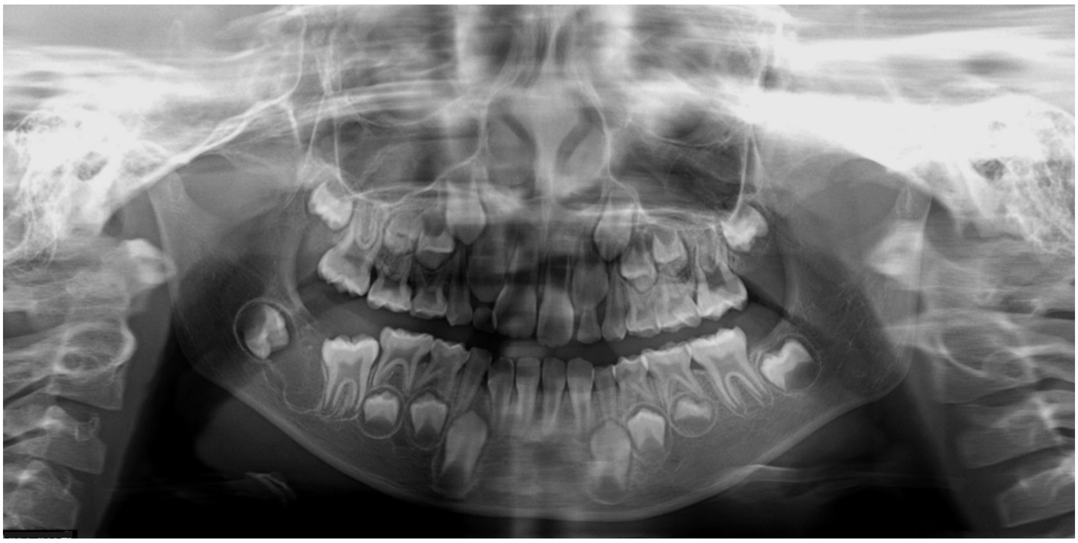

4-ameloblastic fibro-odontoma

Slide test

38. Choose the number according to the lesion location observed in the following panorama and write down its diagnosis.

1) Right maxilla
2) Anterior maxilla
3) Left maxilla
4) Right mandible
5) Anterior mandible
6) Left mandible

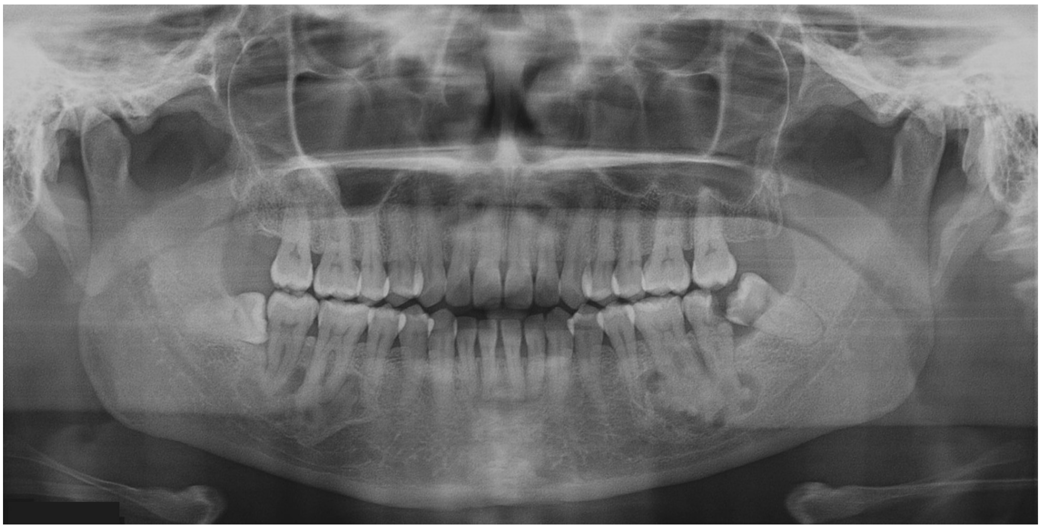

4,6-osseous dysplasia

Slide test

Date: 2023-11-08

39. Choose the number according to the lesion location observed in the following panorama and write down its diagnosis.

1) Right maxilla
2) Anterior maxilla
3) Left maxilla
4) Right mandible
5) Anterior mandible
6) Left mandible

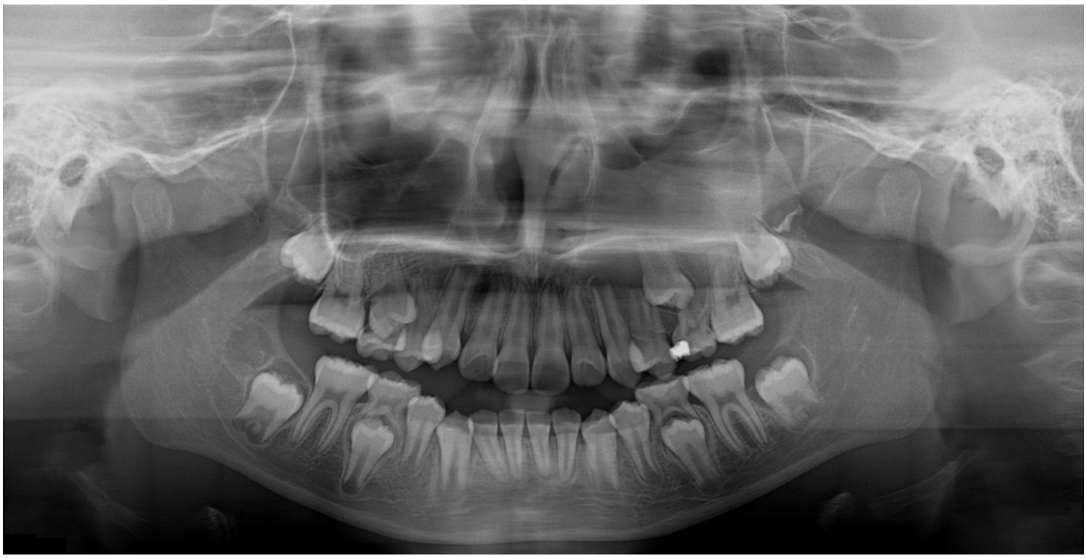

3-fibrous dysplasia

Slide test Date: 2023-11-08

40. Choose the number according to the lesion location observed in the following panorama and write down its diagnosis.

1) Right maxilla
2) Anterior maxilla
3) Left maxilla
4) Right mandible
5) Anterior mandible
6) Left mandible

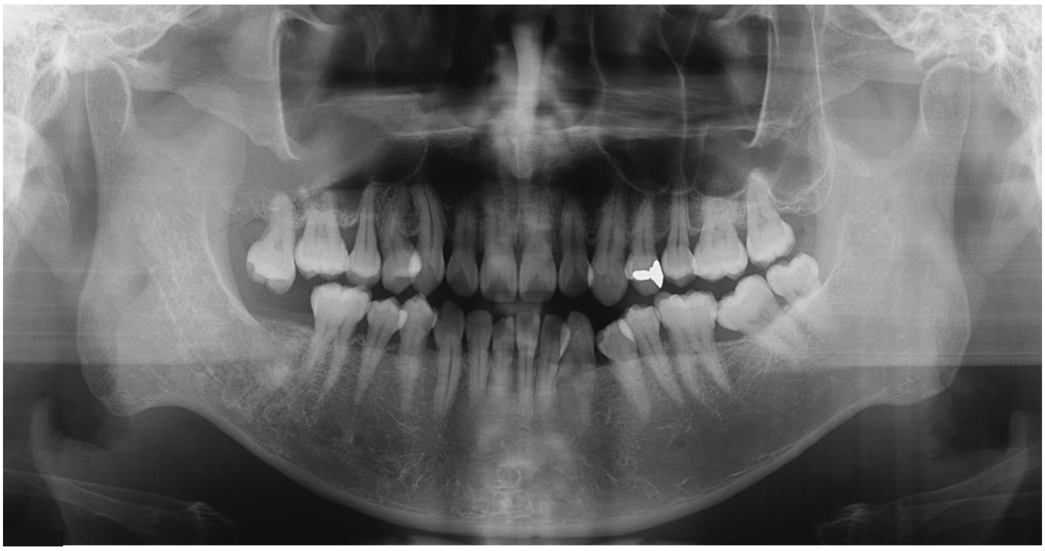

1-malignancy

Slide test

Date: 2023-11-08

41. Choose the number according to the lesion location observed in the following panorama and write down its diagnosis.

1) Right maxilla
2) Anterior maxilla
3) Left maxilla
4) Right mandible
5) Anterior mandible
6) Left mandible

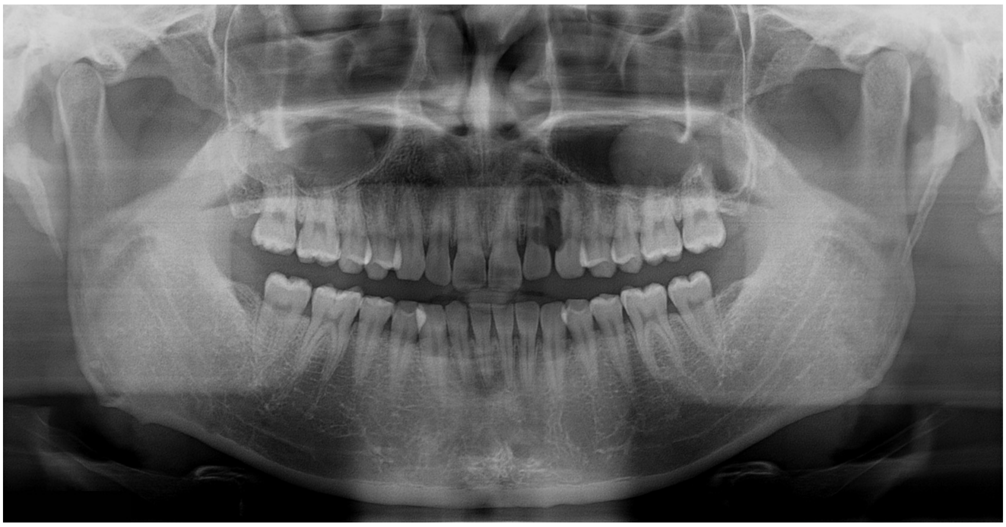

3-central odontogenic fibroma

Slide test

Date: 2023-11-08

42. Choose the number according to the lesion location observed in the following panorama and write down its diagnosis.

1) Right maxilla
2) Anterior maxilla
3) Left maxilla
4) Right mandible
5) Anterior mandible
6) Left mandible

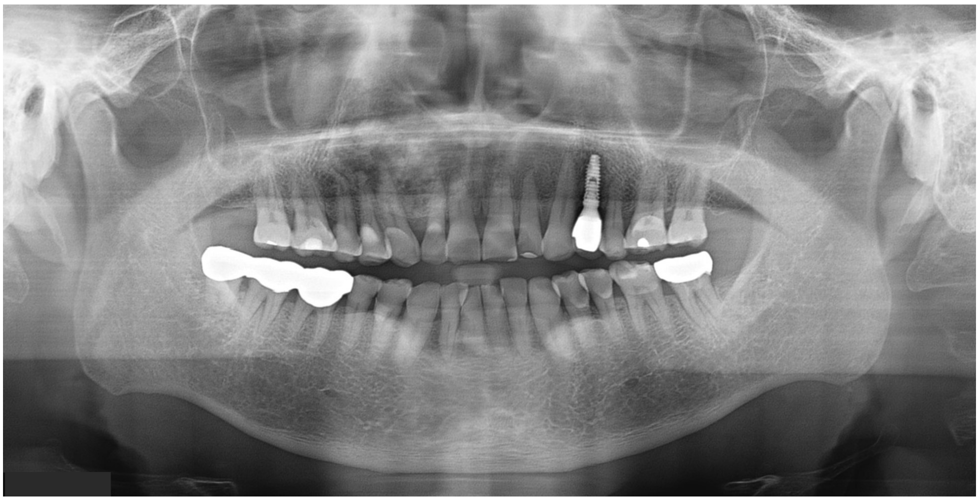

1-ossifying fibroma

Slide test Date: 2023-11-08

43. Choose the number according to the lesion location observed in the following panorama and write down its diagnosis.

1) Right maxilla
2) Anterior maxilla
3) Left maxilla
4) Right mandible
5) Anterior mandible
6) Left mandible

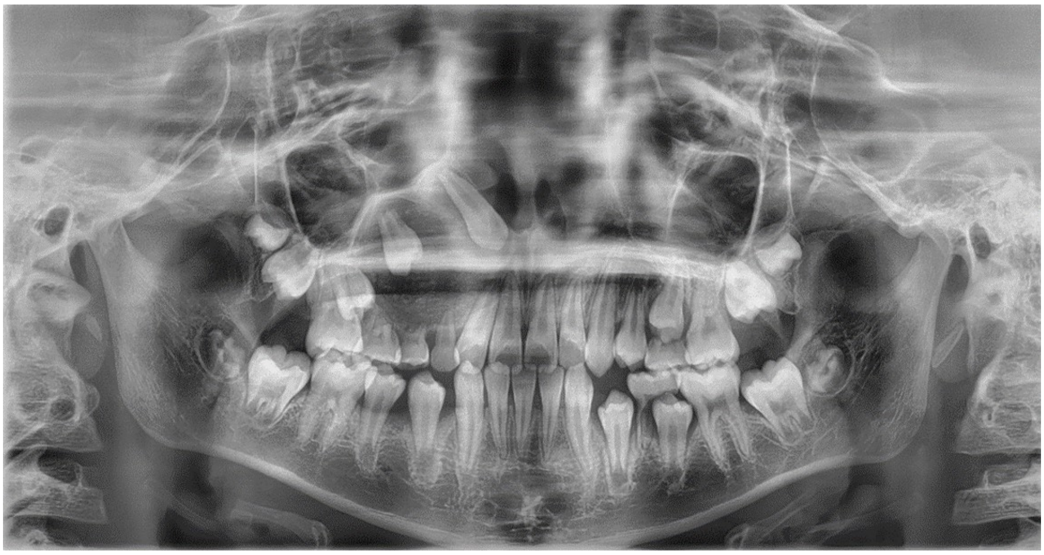

1-adenoid odontogenic tumor

Slide test

Date: 2023-11-08

44. Choose the number according to the lesion location observed in the following panorama and write down its diagnosis.

1) Right maxilla
2) Anterior maxilla
3) Left maxilla
4) Right mandible
5) Anterior mandible
6) Left mandible

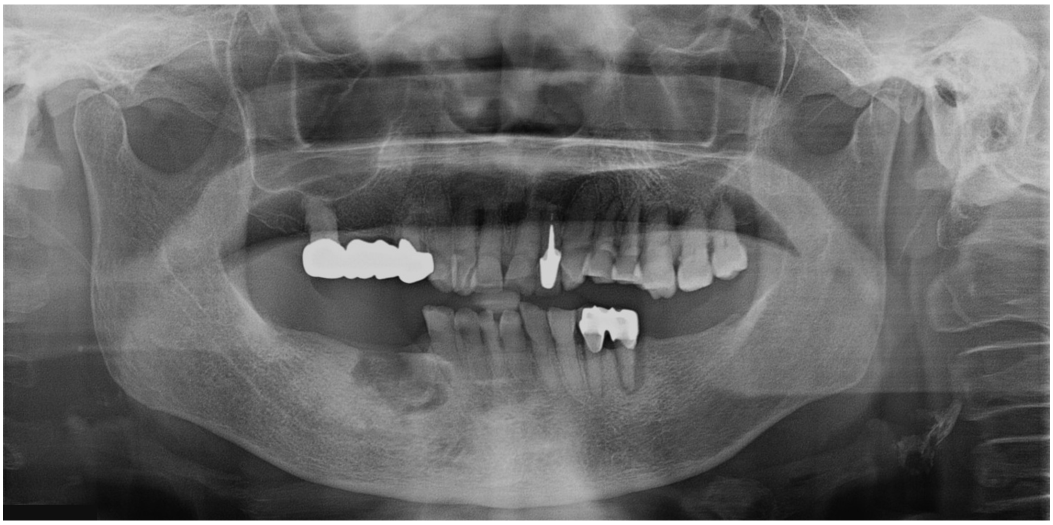

4-osteomyelitis

45. Choose the number according to the lesion location observed in the following panorama and write down its diagnosis.

1) Right maxilla
2) Anterior maxilla
3) Left maxilla
4) Right mandible
5) Anterior mandible
6) Left mandible

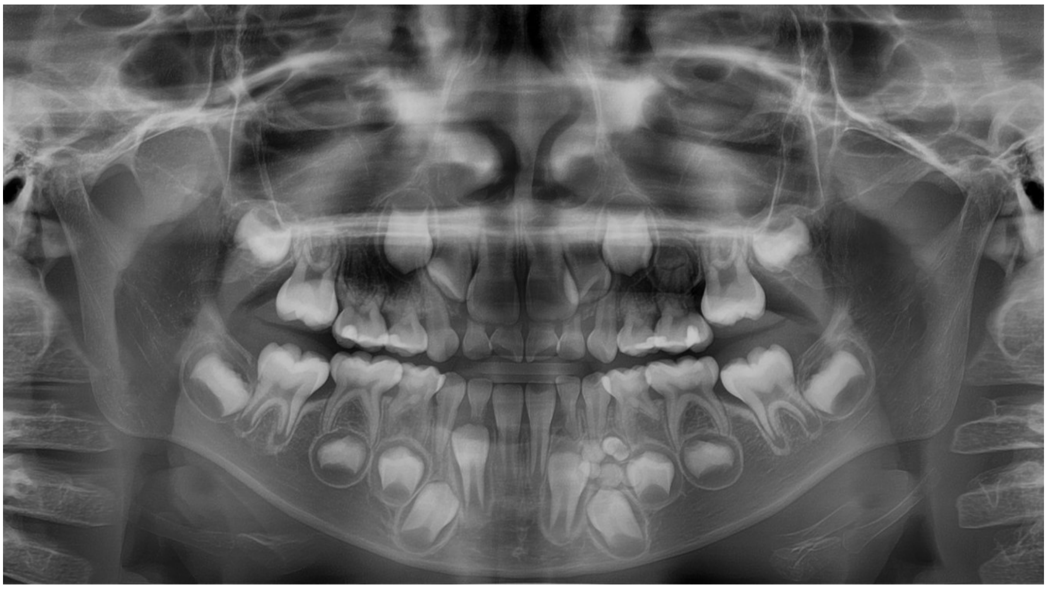

6-odontoma

Slide test

Date: 2023-11-08

46. Choose the number according to the lesion location observed in the following panorama and write down its diagnosis.

1) Right maxilla
2) Anterior maxilla
3) Left maxilla
4) Right mandible
5) Anterior mandible
6) Left mandible

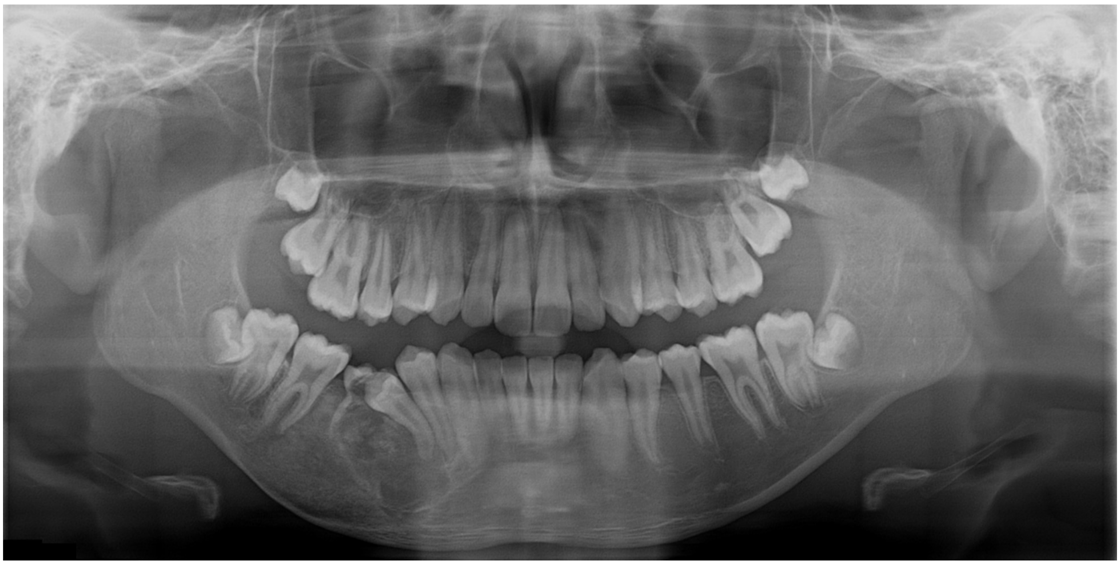

4-ossifying fibroma

47. Choose the number according to the lesion location observed in the following panorama and write down its diagnosis.

1) Right maxilla
2) Anterior maxilla
3) Left maxilla
4) Right mandible
5) Anterior mandible
6) Left mandible

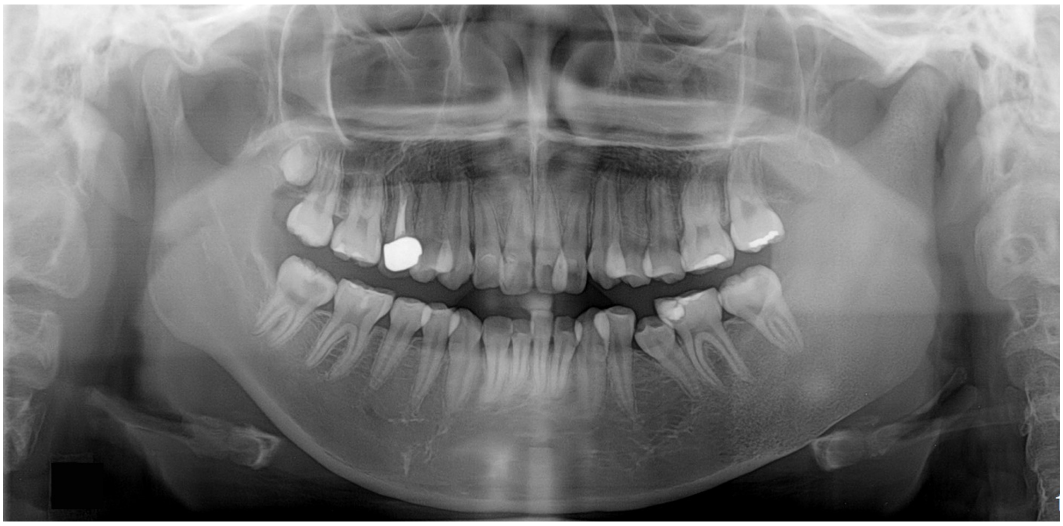

6-fibrous dysplasia

Slide test

Date: 2023-11-08

48. Choose the number according to the lesion location observed in the following panorama and write down its diagnosis.

1) Right maxilla
2) Anterior maxilla
3) Left maxilla
4) Right mandible
5) Anterior mandible
6) Left mandible

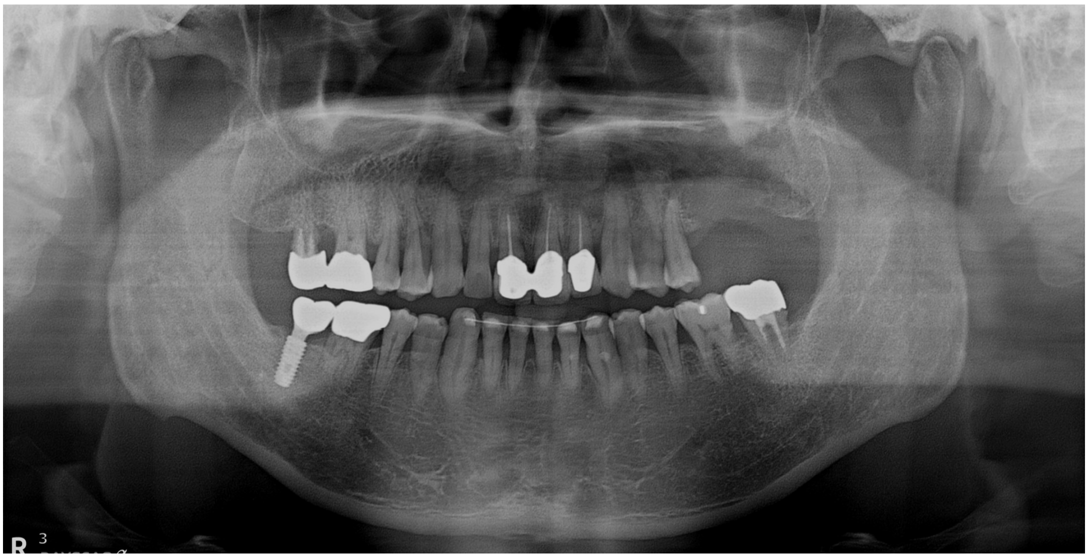

3-osteomyelitis

Slide test Date: 2023-11-08

49. Choose the number according to the lesion location observed in the following panorama and write down its diagnosis.

1) Right maxilla
2) Anterior maxilla
3) Left maxilla
4) Right mandible
5) Anterior mandible
6) Left mandible

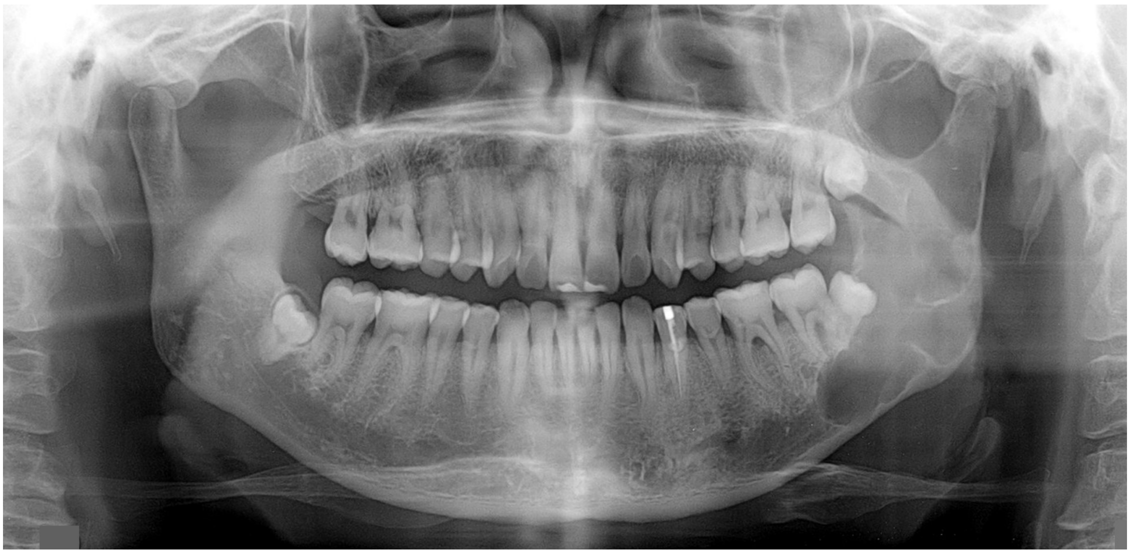

6-odontogenic keratocyst

Slide test

Date: 2023-11-08

50. Choose the number according to the lesion location observed in the following panorama and write down its diagnosis.

1) Right maxilla
2) Anterior maxilla
3) Left maxilla
4) Right mandible
5) Anterior mandible
6) Left mandible

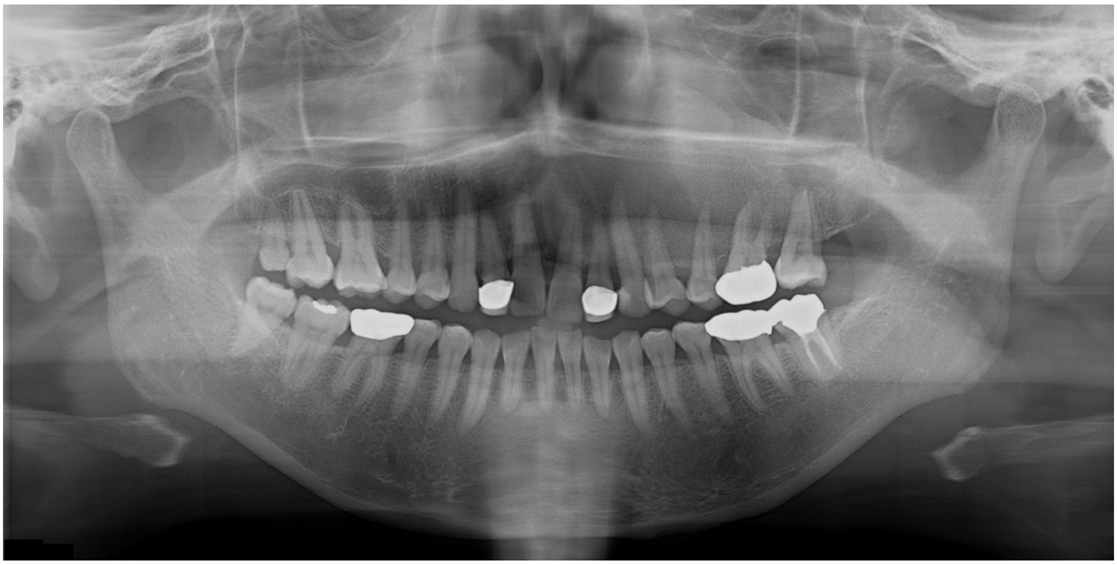

3-fibrous dysplasia
